# Application of spatial transcriptome technologies to neurological diseases

**DOI:** 10.3389/fcell.2023.1142923

**Published:** 2023-03-03

**Authors:** Dongshan Ya, Yingmei Zhang, Qi Cui, Yanlin Jiang, Jiaxin Yang, Ning Tian, Wenjing Xiang, Xiaohui Lin, Qinghua Li, Rujia Liao

**Affiliations:** ^1^ Laboratory of Neuroscience, Affiliated Hospital of Guilin Medical University, Guilin Medical University, Guilin, China; ^2^ Department of Neurology, Affiliated Hospital of Guilin Medical University, Guilin Medical University, Guilin, China; ^3^ Department of Pharmacology, Affiliated Hospital of Guilin Medical University, Guilin Medical University, Guilin, China; ^4^ Guangxi Clinical Research Center for Neurological Diseases, Affiliated Hospital of Guilin Medical University, Guilin Medical University, Guilin, China; ^5^ Department of Neurology ward 2, Guilin People’s Hospital, Guilin, China; ^6^ Department of Geriatrics, Affiliated Hospital of Guilin Medical University, Guilin Medical University, Guilin, China

**Keywords:** spatial transcriptome, neurodegenerative disease, neuropsychiatric illness, stroke, epilepsy

## Abstract

Spatial transcriptome technology acquires gene expression profiles while retaining spatial location information, it displays the gene expression properties of cells *in situ*. Through the investigation of cell heterogeneity, microenvironment, function, and cellular interactions, spatial transcriptome technology can deeply explore the pathogenic mechanisms of cell-type-specific responses and spatial localization in neurological diseases. The present article overviews spatial transcriptome technologies based on microdissection, *in situ* hybridization, *in situ* sequencing, *in situ* capture, and live cell labeling. Each technology is described along with its methods, detection throughput, spatial resolution, benefits, and drawbacks. Furthermore, their applications in neurodegenerative disease, neuropsychiatric illness, stroke and epilepsy are outlined. This information can be used to understand disease mechanisms, pick therapeutic targets, and establish biomarkers.

## Introduction

The human body is a highly complex and delicate system composed of up to trillions of cells. These cells differentiate in a certain microenvironment and develop into organs with unique structures and functions during a set period of time (Liao et al., 2021). When this process is hampered, the organism’s normal physiological functions may be jeopardized. Consequently, cellular heterogeneity and the intricate interactions between cells and the microenvironment contribute to the comprehensive understanding of disease mechanisms at the single-cell level and in the context of tissues. It emphasizes the importance of spatial multi-omics, which explores cellular phenotypes *in situ* and unbiased, amplifying the molecular and spatial structure of tissues and cells, integrating various molecules (DNA, RNA, proteins) and information with four-dimensional resolution (spatial and temporal), provides a detailed cellular atlas from single cells to entire organisms (Lewis et al., 2021). “Spatial transcriptomics (ST)” refers to an omics technique for extracting transcriptome information from cells or tissues that retains spatial information. The application of spatial multi-omics makes it possible for charting cellular heterogeneity, complex tissue structures, and dynamic changes during development and diseases.

It is well-known that RNA levels and protein levels do not always match up. Protein levels are a stronger indicator of cellular activity, particularly in the extracellular matrix and in some cellular states serve as a better proxy for (Moffitt et al., 2022). However, the protein abundance, multiplexing, and throughput limitations of the current imaging and mass spectrometry-based spatial proteomics technologies prevent their widespread use in research diseases. By contrast, high-resolution spatial transcriptomic technologies have been devised to measure hundreds to thousands of mRNAs *in situ*, and these measurements act as faulty substitutes for multiplexing and protein abundance (Paul et al., 2021). Spatial transcriptome technology annotates and maps the spatial distribution of genes while analyzing their expression profiles to precisely characterize and understand their molecular features (Asp et al., 2020). Spatial transcriptome technology provides insight into the exploration of gene expression variations and patterns at the single-cell level or even at the subcellular level, which is a powerful tool to reveal the molecular mechanisms of diseases.

In this review, we summarize the principles, resolution, detection throughput, advantages and limitations of numerous spatial transcriptomics techniques that are now on the market, as well as their enormous potential for application in the study of CNS diseases. We also discuss the field’s current difficulties and prospective future developments. This is a useful reference for the application of spatial transcriptomics technology.

## Classification of space transcriptome techniques

### Spatial transcriptome technology based on microdissection

**Laser capture microdissection coupled with RNA sequencing (LCM-seq)**: LCM is a powerful tool for visualizing and separating morphologically distinct cell subpopulations from heterogeneous tissue specimens (Aguilar-Bravo and Sancho-Bru, 2019; Griesser et al., 2020; Rao et al., 2022). LCM finds regions of cell of interest in tissue sections by microscopy and then excises them with a laser beam to precisely target and isolate individual cells or cell populations for subsequent RNA amplification and transcriptome analysis (Almeida and Turecki, 2022; Bhamidipati et al., 2022). LCM-seq couples LCM with Smart-seq2 RNA sequencing technology to study the expression profile of particular cell (Nichterwitz et al., 2016), directly lyses isolated cells while preserving cell location information. It is suitable for live cells, fresh frozen tissues and formalin-fixed embedded tissues, reproducible and sensitive (Civita et al., 2019; Chang et al., 2021), but the resolution and detection throughput are low.

**Geographical position sequencing (GEO-seq)**: GEO-seq follows LCM-seq based on LCM and single-cell RNA sequencing (scRNA-seq), a technique optimized for tissue collection, cell lysis, RNA isolation and single-cell-based PCR amplification (Chen et al., 2017). These optimized steps maintain the RNA quality of the samples. Also, a specialized bioinformatics pipeline has been developed to analyze GEO-seq data and build a three-dimensional map of the transcriptome. It is able to identify zip-code genes to map cell populations or individual cells to specific locations in the tissue (Stark et al., 2019; Xue et al., 2019). This technique permits the analysis of the transcriptome of only a few cells but is additionally suitable for studying the gene expression profile of rare cells (Yuan et al., 2021). Be that as it may, GEO-seq cannot reach the resolution of a single cell, and it is time-consuming and laborious to construct high-resolution transcriptome profiles based on positional capture of cell samples.

**Tomography sequencing (Tomo-seq)**: Tomo-Seq, a spatially resolved transcriptomic method that combines classical histological sectioning of embryos or tissues with a highly sensitive RNA-sequencing technique (Holler and Junker, 2019). Briefly, the sample is sectioned into thin slices and RNA is extracted from individual slices. Then the CEL-seq2, a scRNA-seq method (Hashimshony et al., 2016), is modified to produce a sequencing library with slice-specific DNA barcodes. That means the cDNA is firstly synthesized by reverse transcription using barcode primers, followed by *in vitro* transcription to linearly amplify the cDNA and prepare a sequencing library. Finally, Illumina sequencing is performed to obtain whole genome expression data with spatial information (Lacraz et al., 2017). Tomo-Seq can be used for whole organisms as well as isolated organs or tissues (Kruse et al., 2016; Naraine et al., 2022), and its detection throughput is large enough to reconstruct the spatial gene expression of thousands of genes. It is an unbiased and systematic way to describe the development of animals, appropriate for developmental biology studies (Boogerd et al., 2022; Iegorova et al., 2022). However, it has the limitation of not providing the spatial resolution of microscopy-based techniques.

**FIGURE 1:**
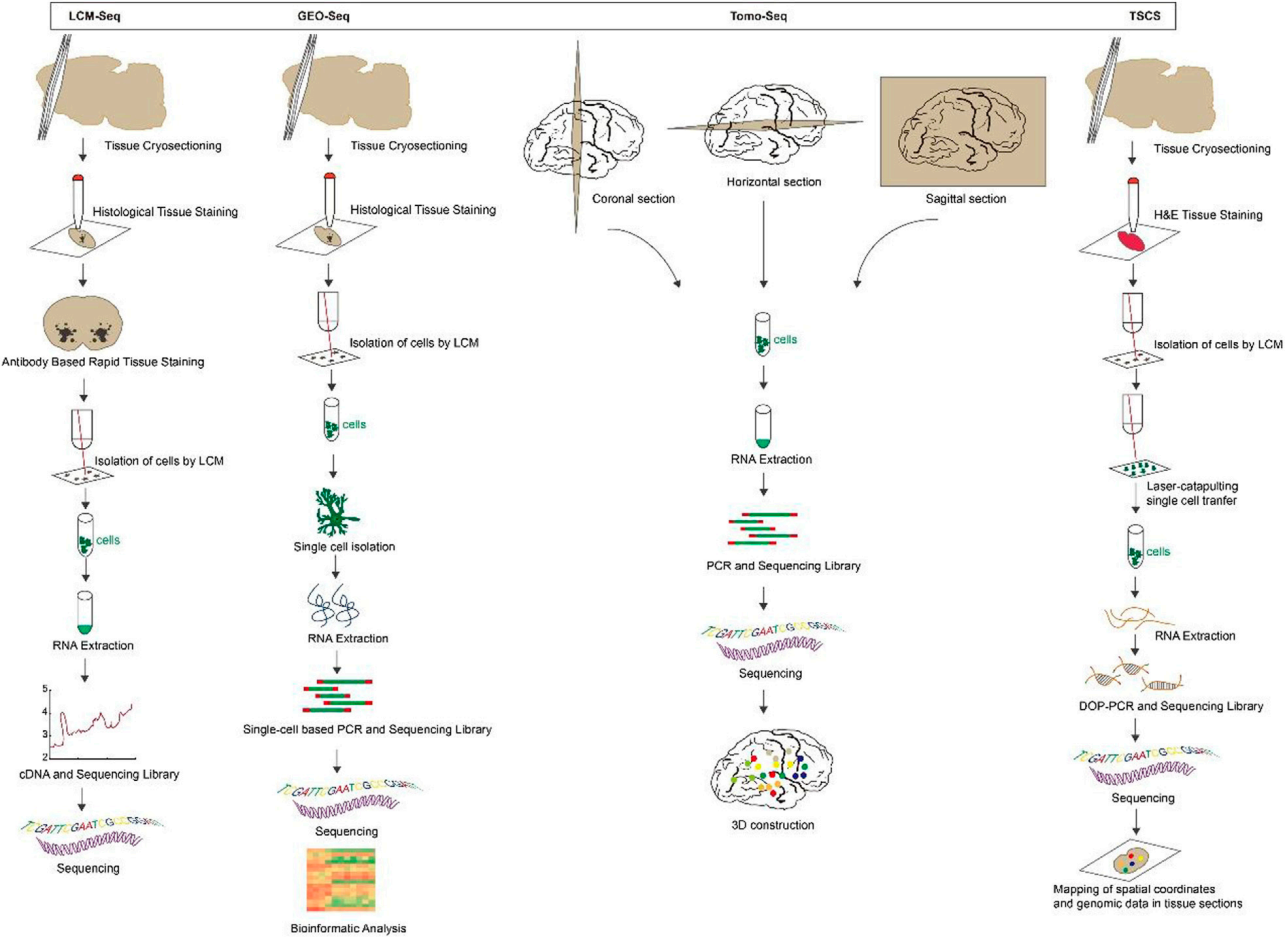
The overview and principle of spatial transcriptome technologies based on microdissection.

**Topographic single cell sequencing (TSCS)**: TSCS is a technology that combines LCM, laser-catapulting, whole-genome-amplification (WGA) and single cell DNA sequencing to measure genomic copy number profiles of single tumor cells while preserving their spatial information (Casasent et al., 2018). To begin with, the sample were sectioned by cryomicrotome, at that point stained with H&E and whole tissue imaging to identify tumor *in situ* and invasive regions. Cells were isolated by a 1-micron laser and transferred to collection tubes by laser-catapulting. After that, WGA and next-generation sequencing (NGS) were performed, the data was demultiplexed by cell library barcode and prepared to calculate genomic duplicate number profiles. At last, single-cell genomic information is mapped to spatial coordinates, not only for the genomic copy number profiles of individual tumor cells, but to depict the topographic organization of different clonal genotypes in the tissue sections (Wan et al., 2003; Ren et al., 2018; Lahnemann et al., 2020). Figure 1.

### Spatial transcriptome technology based on *in situ* hybridization

**Single-molecule fluorescence *in situ* hybridization (smFISH)**: Individual mRNA molecules in a fixed cell or tissue are imaged by hybridization with a few particularly labeled short DNA oligonucleotide probes which are complementary to the sequence of the target mRNA, and each probe are coupled to some fluorescent dyes. Imaging is then performed by recognizing the fluorescent signal displayed by the probes (Imbert et al., 2022; Piskadlo et al., 2022). This method is called smFISH that enables subcellular localization and quantification of mRNA molecules in individual cells (Kocks et al., 2018). In addition, multi-color, single-molecule fluorescence *in situ* hybridization permits simultaneous examination of several different transcripts, but it is troublesome to recognize a large number of colors by optical microscopy (Wang, 2019). Based on probe design, smFISH is classified as: short probes labeled with multiple fluorophores, short probes labeled with single fluorophores, short probes with modified backbones and signal amplification of single molecule probes (Kwon, 2013). smFISH is a powerful single-cell transcriptional profiling method for any organism, cell culture and tissue sectioning (Haimovich and Gerst, 2018). It is specific and sensitive enough to quantify numerous RNAs at the single-molecule level and to obtain spatial information about the localization of RNA in the cell without signal amplification steps. It is presently available to measure 10–30 different RNA species in a single cell simultaneously by color-based barcoding or sequential hybridization utilizing combinatorial markers (Biancalani et al., 2021).

**Sequential fluorescence *in situ* hybridization (seqFISH)**: seqFISH is a temporal barcoding method that uses a limited number of fluorophores scaled exponentially with time to address the issue of fluorophore limitation on smFISH. Specifically, sequential rounds of hybridization, imaging, and probe stripping are performed on mRNA in fixed cells (Lubeck et al., 2014). Each round of hybridization uses the same FISH probe labeled with a single fluorophore, and the next round replaces the FISH probe with another color. Multiple rounds of hybridization give the mRNA a distinct temporal barcode, which is then decoded by aligning the images of the barcode hybridization under super-resolution microscopy (Shah et al., 2016; Lohoff et al., 2022). The number of barcodes available scales as F^N^, where F is the number of fluorophores and N is the number of rounds of hybridization. Theoretically, 8 rounds of hybridization using 4 dyes can cover the entire transcriptome (Shah et al., 2017; Kim et al., 2019). As the number of hybridizations increases, the signal loss due to mis-hybridization also remarkably increases (Shah et al., 2016).

**Multiplexed error-robustness fluorescence *in situ* hybridization (MERFISH)**: The imaging method MERFISH can assess the spatial localization and copy number of thousands of mRNAs in a single cell utilizing combinatorial labeling and error-resistant barcodes (Chen et al., 2015; Zhang et al., 2021). During imaging, the presence or absence of color at each site is indicated by “1”or “0”using a N-bit binary coding combination marker. After N rounds of hybridization, 2^N^-1 mRNA species can be identified. However, as the number of detected sites increases, the frequency of misreading “1”as “0”or “0”as “1”increases. The misidentification of mRNA occurs as a result of an increase in the frequency of measurement errors (Fang et al., 2022). As a result, MERFISH employs an error-correcting or error-robust coding scheme that establishes a minimum Hamming distance (the Hamming distance is the number of misidentified sites in a binary barcode). This ensures that barcodes are only misidentified when multiple errors occur simultaneously, thereby decreasing the rate of misidentification (Moffitt and Zhuang, 2016; Xia et al., 2019). Without amplification bias, MERFISH is able to simultaneously image and identify hundreds to thousands of distinct mRNA species within the primary spatial context of individual cells. This enables high-throughput differential gene expression and covariance analysis (Lu et al., 2021; Liu et al., 2023).

**Sequential fluorescence *in situ* hybridization + (seqFISH+)**: seqFISH+ was further developed, which uses sequential hybridizations and standard confocal microscope imaging to achieve super-resolution imaging and multiplexing of 10,000 genes in single cells (Eng et al., 2019). seqFISH + extends the barcode base palette from the 4-5 colors used in seqFISH to a larger “pseudocolor” palette that uses 60 pseudo-color channels to encode barcodes, effectively diluting the mRNA molecules into 60 separate images, positioning each mRNA site below the diffraction limits before reconstructing super-resolution images. In order to avoid chromatic aberrations between channels, the 60 pseudo-colors are divided into three fluorescent channels, and barcodes are only generated within each channel. By repeating this pseudocolor imaging four times (with one round used for error correction), 20^3^ = 8,000 genes can be barcoded per channel, for a total of 24,000 genes (Takei et al., 2021; Bao et al., 2022). When compared to seqFISH, the imaging time of seqFISH + technology is significantly shorter. In addition, seqFISH + reduces the number of errors caused by sequential hybridization by overcoming optical crowding, making use of a large number of pseudo-colors, and employing shorter barcodes.

**FIGURE 2:**
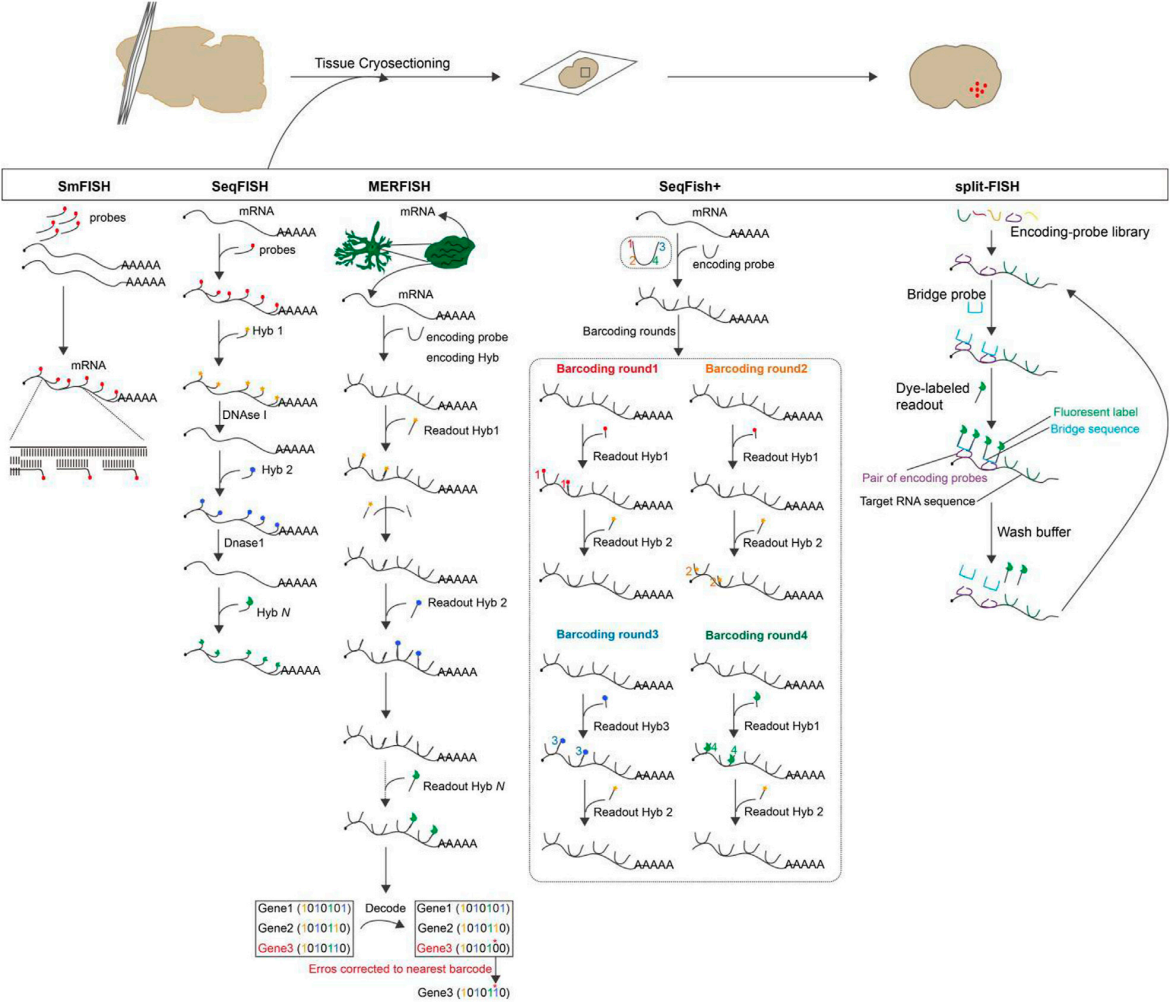
The overview and principle of spatial transcriptome technologies based on *in situ* hybridization.

**split-FISH**: split-FISH is a multiplex fluorescent *in situ* hybridization method that uses split probes to improve the specificity of mRNA detection. A bridge sequence is designed between a pair of adjacent encoding probes. It is only when the pair of encoding probes hybridized at adjacent positions on target mRNA that there is sufficient complementary base pairing for the bridge probe to bind effectively. The bridge probe does not bind to either encode probe stably (Goh et al., 2020). A mRNA fluorescence signal is then produced when the fluorescently labeled readout probe hybridizes with the bridge probe. False positives are reduced, background fluorescence caused by the probe binding off-target is reduced, and accurate mRNA analysis in unclean tissue is made possible by Split-FISH (Takeuchi et al., 2012; Togashi et al., 2018). Figure 2.

### *In situ* sequencing-based spatial transcriptome technology

**Fluorescent *in situ* sequencing (FISSEQ)**: FISSEQ is a non-targeted amplification detection method for gene expression profiling in fixed cells or tissues by converting mRNA into cross-linked cDNA amplicons and using SOLiD sequencing by ligation (Lee et al., 2015). Firstly, mRNA is reversing transcribed (RT) in fixed cells with labeled random hexamers. To prevent diffusion of cDNA fragments, primary amines are introduced into cDNA fragments by aminoallyl DUTP during RT to form amine-modified cDNA, and then the cDNA is immobilized on a cellular protein matrix by cross-linking primary amines with BS(PEG)9. Each cDNA is linearly amplified by rolling circle amplification (RCA) into a single molecule containing multiple copies of the original cDNA sequence, and BS(PEG)9 is used to cross-link the RCA amplicons. Subsequently, SOLiD sequencing by ligation is used for imaging and sequencing (Lee, 2017; Wang et al., 2021). FISSEQ is a transcriptome-wide unbiased *in situ* sequencing technique that overcomes optical resolution’s limitations and noisy signals’ impact on single-molecule detection (Lee et al., 2014; Nguyen et al., 2020). However, its sensitivity is low.

**Barcode *in situ* targeted sequencing (Baristaseq)**: Baristaseq is an improved version of padlock probe-based *in situ* barcode sequencing technology that combines Illumina sequencing by synthesis (SBS) with a five-fold increase in detection efficiency (Chen et al., 2018). Barcodes can be used to uniquely label individual cells within a population, and as the sequence length increases, their diversity increases exponentially. These barcodes can be quickly distinguished owing to the high spatial resolution and high throughput of *in situ* sequencing. Furthermore, phusion DNA polymerase, which lacks strand displacement activity, is used in Baristaseq instead of Stoffel fragments, resulting in increased sensitivity and specificity. Additionally, Baristaseq employing Illumina sequencing chemistry has a lower background signal than SOLiD sequencing and is extremely accurate for cellular barcode sequencing (Chen et al., 2019; Zhang et al., 2022a).

**Spatially resolved transcript amplicon readout mapping (STARmap)**: STARmap is a technology for 3D intact-tissue RNA sequencing, which integrates hydrogel-tissue chemistry (HTC), targeted signal amplification, and *in situ* sequencing (Wang et al., 2018). The signal is target-specific amplified by a method called SNAIL, where the probe is enzymatically replicated as a cDNA amplicon only if the primer and padlock probe hybridize to the same mRNA. After processing, the amplicons are stably cross-linked with tissue hydrogels to create tissue hydrogel complexes. To enable multiplexed gene detection, each SNAIL probe is designed with a gene-specific identifier. A sequencing of error reduction by dynamic annealing and ligation (SEDAL) method was designed to eliminate errors generated by sequencing, using a reading probe to decode the bases and a fluorescent probe to convert the decoded sequence information into a fluorescent signal. These two short probes only ligate to form a stable product for imaging when they are perfectly matched to the target DNA transient binding. Three-dimensional highly multiplexed mRNA quantification reveals spatial gene expression and cell type information (Kebschull et al., 2020; Biancalani et al., 2021). The advantage of STARmap is that SNAIL lessens the limitations of reverse transcription on *in situ* sequencing efficiency. Secondly, hydrogel-based tissue transformation technology synthesizes polymers *in situ*, enabling high-resolution imaging and analysis of tissues while preserving and extracting bimolecular information, which improves mechanical stability, reduces background, and increases optical transparency (Bao et al., 2022). SEDAL has a much smaller background than SOLiD. In addition, to cut down on residual errors caused by high-density speckle imaging, a two-base coding scheme was developed and implemented for SEDAL.

**FIGURE 3:**
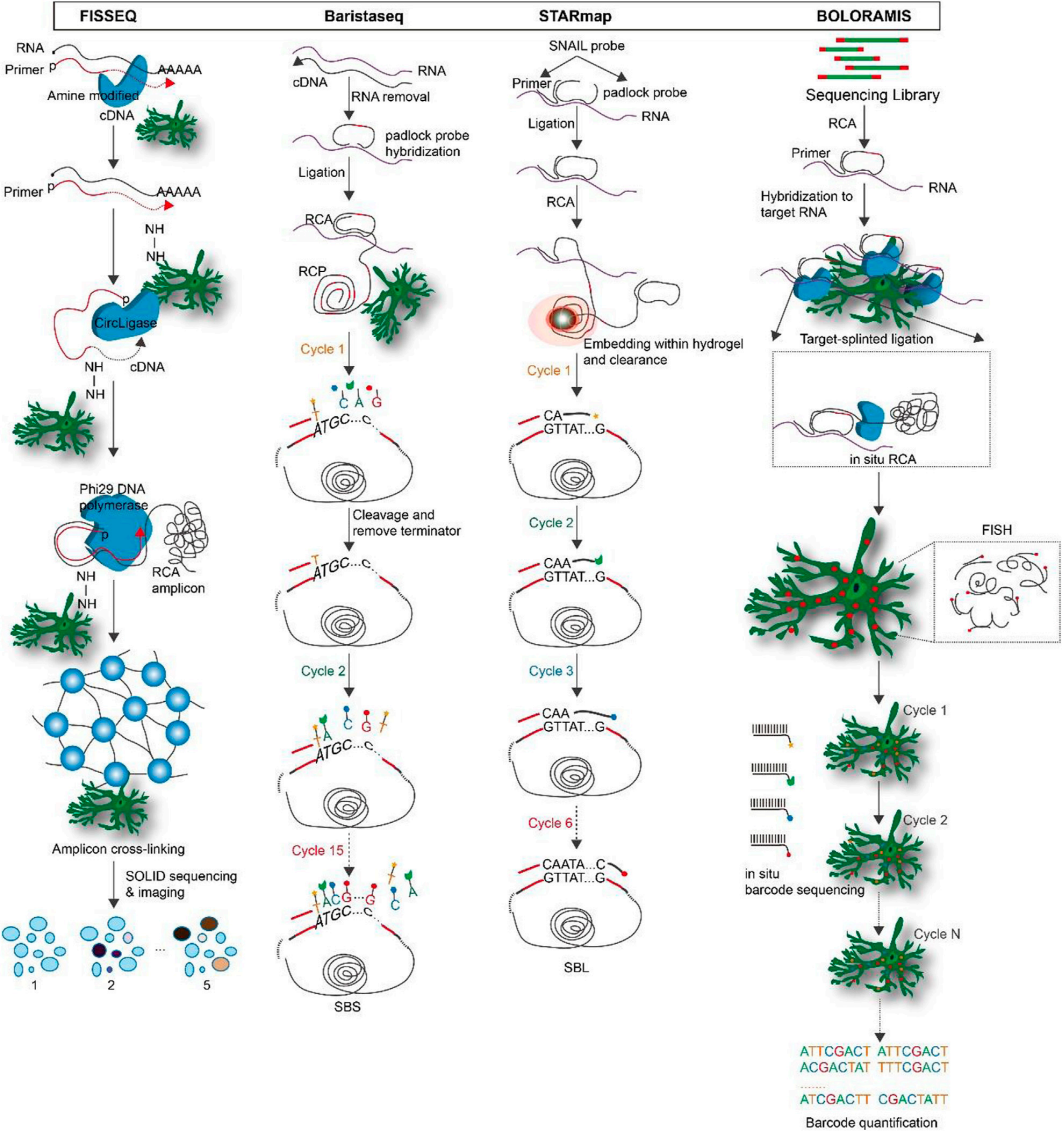
The overview and principle of spatial transcriptome technologies based on *in situ* hybridization.

**Barcoded oligonucleotides ligated on RNA amplified for multiplexed and parallel *insitu* analyses (BOLORAMIS)**: This mRNA detection technique by performing multiple parallel *in situ* analyses of barcoded oligonucleotides attached to mRNA without RT for spatially resolved, targeted, *in situ* mRNA identification of one or more targets (Liu et al., 2021). BOLORAMIS is based on combinatorial molecular indexing combined with direct mRNA-dependent ligation and clonal amplification of barcoded padlock probes. After cell fixation and permeabilization, probes are added so that they hybridize directly to mRNA molecules. This eliminates the need for RT to convert mRNA to cDNA. The probes are then ligated with SplintR ligase and RCA is performed to generate amplicons. After that, the amplicons are cross-linked to the cell matrix to prevent translocation and the barcodes are detected by fluorescence *in situ* hybridization (FISH) or sequenced *in situ* to obtain genetic information about the bases they encode. BOLORAMIS is more sensitive than non-targeting methods like FISSEQ, Slide-seq, and high-definition spatial transcriptomic (HDST) because it targets specific genes of interest in a highly multiplexed manner (Kleino et al., 2022; Moffitt et al., 2022). By employing SplintR ligase, BOLORAMIS eliminates the need for RT in comparison to *in situ* padlock probe methods, thereby reducing the potential for detection bias and the cost of experiments associated. BOLORAMIS has the shortest blot required for *in situ* transcript detection, i.e., 25 nt, when compared to other RT-free methods like MERFISH, seqFISH+, and STARmap (Zhang et al., 2022a; Moses and Pachter, 2022). Figure 3.

### *In situ* capture-based spatial transcriptome technology

**Spatial transcriptomics (ST):** Spatial transcriptomics is a technique for visualizing and quantifying mRNA at spatial resolution through barcode microarray slices. Tissues are placed on oligonucleotide microarrays slide (slide surfaces are shown as spots), and each array features contains unique positional barcode, unique molecular identifier (UMI), poly(d)T oligonucleotides and sequencing primer. Then the tissues are fixed, stained with H&E, and imaged. After permeabilization of tissue sections, poly(d)T captures mRNA and visualizes cDNA synthesis with fluorescently labeled nucleotides. After tissue is enzymatically removed, the cDNA containing UMI information and spatial barcodes is obtained. NGS is used to later create and sequence amplification-based sequencing libraries. For the purpose of visualization and analysis, the tissue images are aligned with array features, and the RNA-seq data is classified using spatial barcodes (Stahl et al., 2016; Rao et al., 2021). The integration of histological imaging and gene expression profiling is a primary feature of ST. Histological imaging is performed by conventional staining schemes to capture morphological features, whereas expression profiling is carried out through processing and sequencing of spatially barcoded cDNA (Moncada et al., 2020; Srivatsan et al., 2021). The first generation of ST microarrays consisted of ∼1,000 spots, each with a diameter of 100 μm, and thus provided an averaged transcriptomic profile from a mixture of tens of cells. The ST technology was further developed and commercialized by 10x Genomics, which enabled the detection of one to 10 cells with increased sensitivity and throughput (∼5,000 spots with a spot diameter of 55 mm) (Stahl et al., 2016; Larsson et al., 2021).

**Slide-Seq**: Slide-Seq is a measurement technique for high-resolution genome-wide expression analysis that uses beads combined with spatially indexed barcodes to infer the location of mRNA through spatial mapping (Wang et al., 2021). SOLiD sequencing chemistry can be used to determine the DNA barcode sequence on each bead, which is placed on a single layer of rubber-coated glass coverslip (Rodriques et al., 2019). Fresh frozen tissue sections are transferred to the surface of dry beads, and the beads capture the mRNA released from the tissue to prepare a barcoded RNA-seq library. The library is finally decoded by SOLiD sequencing chemistry to identify the spatial location of the mRNA. Although slide-seq has a spatial resolution of 10 μm, its application is constrained by its low transcript detection sensitivity. Slide-seqV2 is created as a result of subsequent advancements in array indexing, bead synthesis, library generation, and mRNA capture efficiency that are approximately ten times greater than Slide-seq’s (Chen et al., 2021; Stickels et al., 2021).

**High-definition spatial transcriptomic (HDST)**: Similar to Slide-seq, HDST uses spatial barcode bead arrays to capture mRNA from tissue sections for 2 μm resolution spatial gene expression analysis. 2,839,865 individually barcoded beads, each containing a different pool of spatial barcodes, unique molecular identifiers, and poly(d)T, are randomly placed into hexagonal arrays of 2 μm wells. The positions of the beads are then decoded through multiple rounds of sequential hybridization (Vickovic et al., 2019). Frozen tissue sections are placed on decoded slides, after staining and imaging, mRNA released from frozen tissue sections is captured and reverse transcribed, and then analyzed by RNA-seq. This method has subcellular resolution, but it is difficult to use in a lot of studies due to the random distribution of beads on the array, each of which needs to be individually decoded for surface barcodes before use (Wang et al., 2021).

**Deterministic barcoding in tissue for spatial omics sequencing (DBiT-seq)**: DBiT-seq is a multi-omics sequencing technology based on microfluidic technology to introduce barcodes into tissue sections, therefore obtain transcriptomic and proteomic information at near single-cell resolution (Su et al., 2021). In practice, combined DNA barcodes (A and B) are introduced into tissue sections using microfluidic chips, leading to *in situ* reverse transcription of mRNAs to generate cDNAs containing the combined barcodes (Liu et al., 2020). Subsequently, the combined barcodes are joined at the intersection to produce a two-dimensional image of the tissue. Tissue slides are stained with a mixture of antibody-derived DNA tags (ADTs) to identify proteins prior to the introduction of barcodes. After forming a spatially barcoded tissue image, the tissue is digested, the spatially barcoded cDNAs are recovered for PCR amplification, an NGS sequencing library is constructed, and finally the corresponding transcripts and protein barcodes are detected using paired-end NGS sequencing to reconstruct the spatial expression profile (Miller et al., 2022).

**Seq-Scope**: Seq-Scope is a spatial transcriptomic technology that combines spatial barcodes and Illumina sequencing to achieve high resolution, displaying the organization of the transcriptome at the tissue, cellular and even subcellular levels (Cho et al., 2021). There are two main sections to the Seq-scope. The first step is to generate a physical array of RNA capture molecules encoded by high-definition map coordinate identifiers (HDMI) and its corresponding barcodes using the MiSeq method of the Illumina sequencing platform. The second step involves capturing mRNAs released by the tissue from the physical array, reverse transcribed to obtain cDNA, followed by the synthesis of secondary strands using random primers, PCR collection and preparation of secondary strand sequencing libraries, and finally a paired-end sequencing to obtain cDNA sequences and their matching HDMI barcodes with spatial location information (Do et al., 2022). Seq-Scope has ultra-high resolution and high efficiency mRNA capture rate. The current limitation of Seq-Scope is that it can only be used to study the transcriptome of poly-A tagged genes.

**FIGURE 4:**
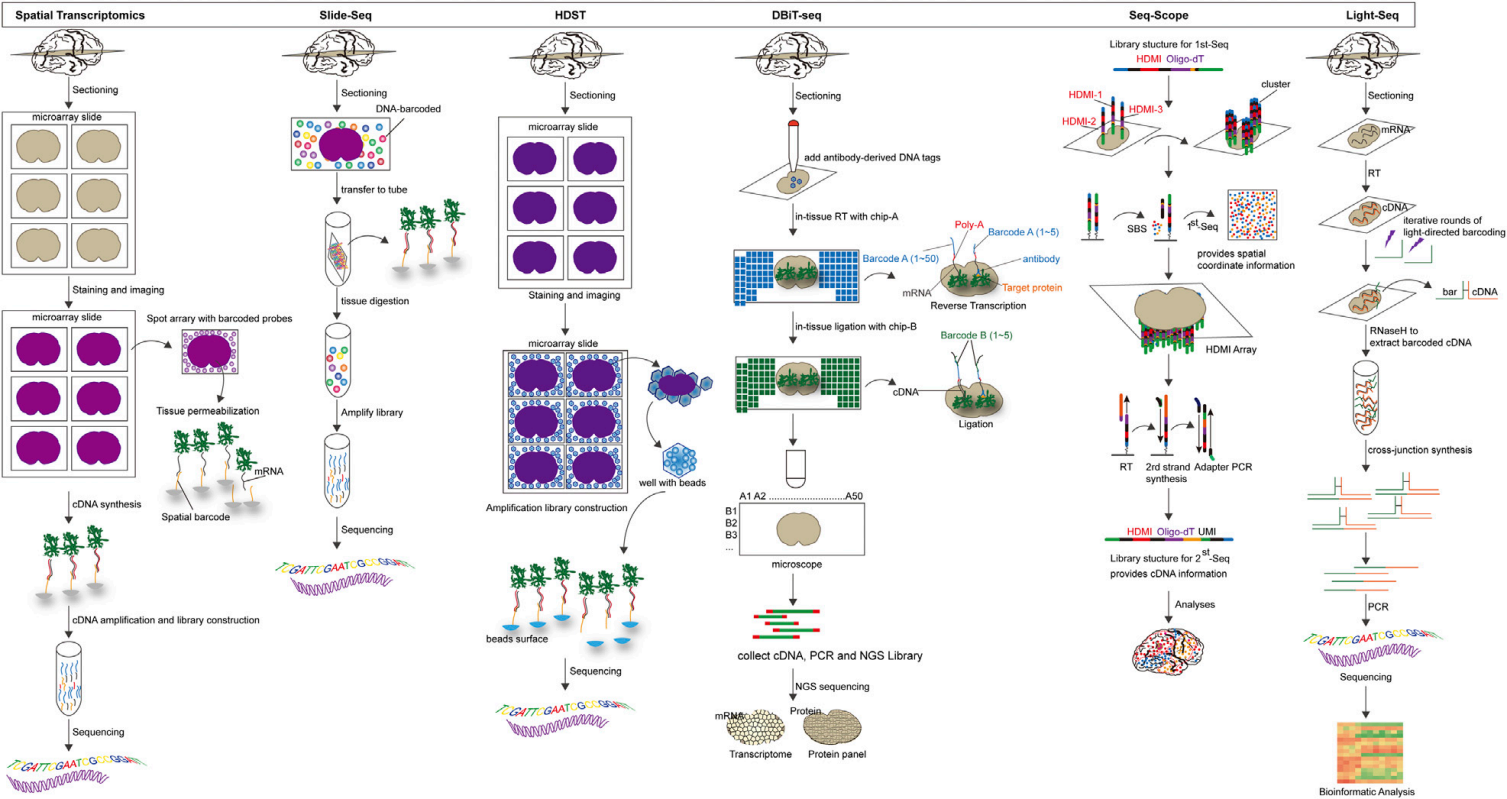
The overview and principle of *in situ* capture-based spatial transcriptome technology.

**Light-Seq**: Light-seq is a method for sequencing in fixed cells and tissues using photoconductive DNA barcoding (Kishi et al., 2022). On fixed and permeabilized cell or tissue sections, the cDNA is first synthesized by *in situ* RT, and then DNA barcodes containing ultrafast photo-crosslinkers are bound to the cDNA by UV irradiation. A cross-linking synthesis reaction incorporates the DNA barcodes and cDNA into new DNA single strands to produce a combined, spatially indexable sequencing library. Next, NGS is used to enable imaging and whole transcriptome sequencing of selected cells in fixed samples. Figure 4.

### Space transcriptome technology based on living cell markers

**Transcriptome *in vivo* analysis (TIVA)**: TIVA is an ideal method for fluorescently labeling cells in living tissue, selectively activating and isolating mRNA from cells of interest, and retaining information about the spatial location of individual cells (Yeldell et al., 2020). The TIVA-tag is a versatile light-activated mRNA capture molecule that penetrates the cell with the help of cell-penetrating peptide (CPP) (Lovatt et al., 2014). The fluorescently labeled TIVA-tag precisely directs us to the target mRNA. It is then selectively photo-activated by the laser. The affinity tag at the end of the mRNA is purified to form a TIVA-mRNA hybrid, which is then eluted off and the captured mRNA can be used for RNA-seq transcriptome analysis. TIVA is a high-resolution transcriptome method that can capture mRNA of varying sizes and abundances from single cells in living sections without causing significant cellular damage and is suitable for living cells or living tissues (Schneider and Hackenberger, 2017).

**Zipseq**: ZipSeq is a technique that uses pattern-specific illumination and light-caged oligonucleotides to label DNA barcodes (Zipcodes) onto the surface of living cells within intact tissues, linking scRNA-seq with spatial dimension and real-time phenotypic analysis (Hu et al., 2020). Specifically, double-stranded DNA is anchored to the cell membrane of the region of interest by high-affinity antibodies or lipid-modified oligonucleotides. Under specific illumination, cage-locked sequences with Zipcode on the double-stranded DNA are released to hybridize with fluorescent probes. Afterwards, in combination with high-throughput sequencing, makes it possible to image and spatially code the transcriptome of the region of interest (Xu et al., 2014).

**FIGURE 5:**
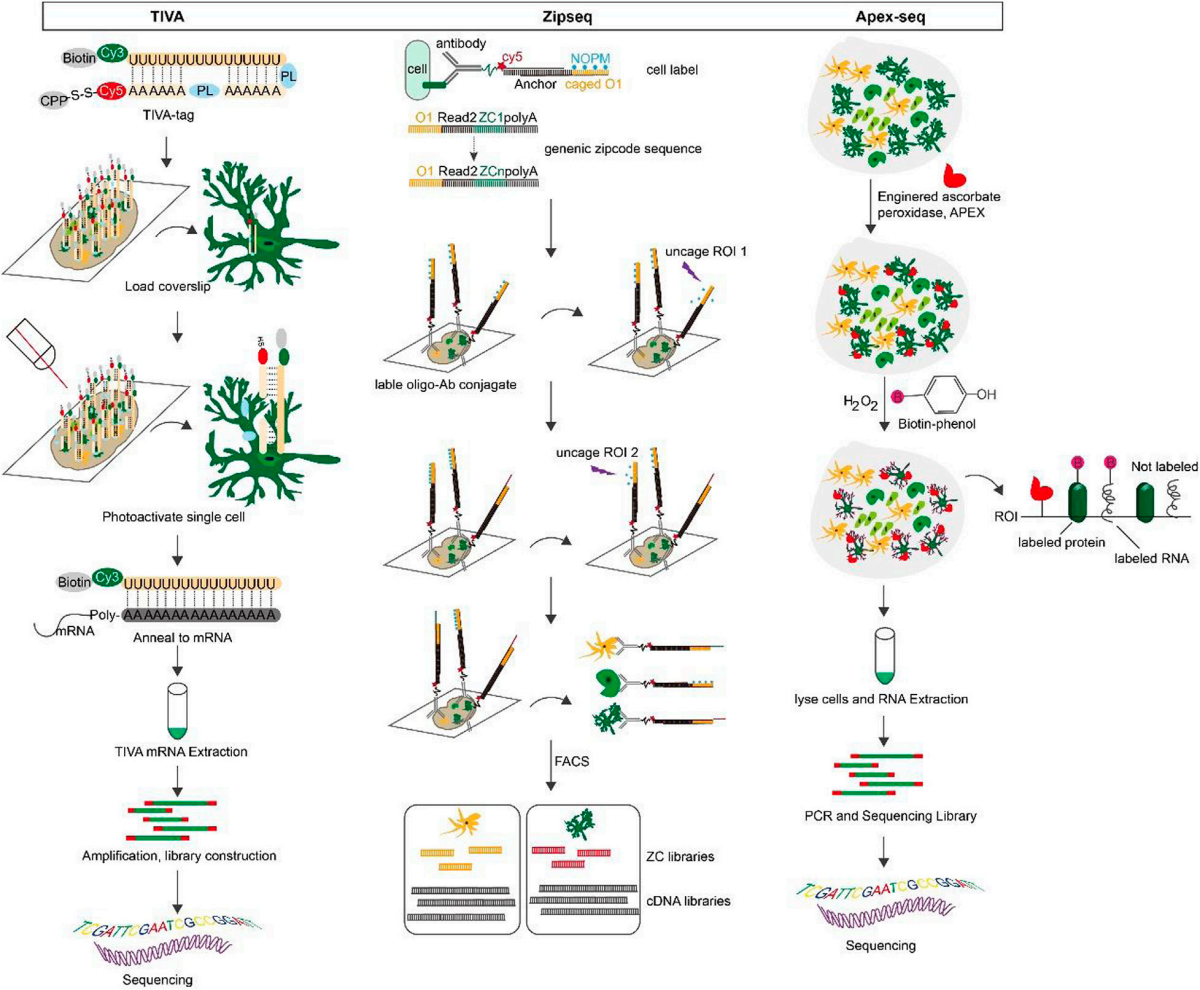
The overview and principle of space transcriptome technology based on living cell markers.

**APex-seq**: APex-seq is a sequencing method that uses the peroxidase APEX2 to target cells in a cellular region of interest within living cells (Fazal et al., 2019). APEX-seq can generate nanometer-resolution spatial maps of the transcriptome in various subcellular regions, precisely characterize the spatial organization of mRNAs, reveal the localization patterns of different types of mRNAs and transcriptional isoforms, and link mRNA localization to genome structure, protein localization, and local translation mechanisms (Padron et al., 2019). Additionally, “non-purifiable” structures like the nuclear layer and mitochondrial outer membrane can be analyzed with APEX-seq; it can also detect lncRNAs, antisense RNAs, and untranslated mRNAs not bound by ribosomes, but it cannot provide single-cell information (Wu et al., 2021; Li et al., 2022). Figure 5.

## Application of spatial transcriptome technology in nervous system diseases

### Neurodegenerative disease

Spatial transcriptomics not only provides information on gene expression, but also spatially locates the pathological mechanisms of disease based on spatial location information (Liu et al., 2022). One study found through spatial transcriptomics that the center of mixed active/inactive injuries in postmortem multiple sclerosis (MS) brain tissues from humans as well as the acute stage of experimental autoimmune encephalomyelitis development in mice exhibit the greatest reduction in autophagy-related (ATG) gene expression (Misrielal et al., 2022). The results help to understand the role of autophagy in different stages of MS pathology, and measuring ATG expression can help to assess disease severity and progression. Another study used spatial transcriptome technology with high spatial resolution to identify the heterogeneous distribution of neurodegeneration in the middle gray matter of the brains of MS patients (Kaufmann et al., 2022). Regional data was also used to infer the temporal evolutionary pattern of neurodegeneration and to investigate the mechanisms of early intercellular communication in MS neurodegeneration in order to provide the most appropriate therapeutic intervention.

Motor neuron degeneration in amyotrophic lateral sclerosis (ALS) results in muscle denervation atrophy (Lepine et al., 2022). Therefore, it is crucial to use spatial transcriptomics to collect and examine changes in ALS gene expression in order to identify cellular subpopulations associated with each stage of the disease process and to investigate the underlying molecular mechanisms that cause and sustain the disease. Early microglia gene expression changes in ALS are mediated by TREM2 and TYROBP, according to a study that used spatial transcriptomics (ST) to examine gene expression changes in postmortem tissues of ALS patients and mouse models (Maniatis et al., 2019). Gene expression signatures of regional astrocyte populations with distinct, disease-related spatio-temporal dynamics were discovered through spatial co-expression analysis. Additionally, this study revealed a number of transcriptional pathways shared by human postmortem spinal cord models and mouse models of ALS, providing data and information for subsequent research. The spatial transcriptome of human ALS cortical tissue was examined in another study using ST and Basescope technology (Gregory et al., 2020), and ST found 16 candidate transcripts with differential expression that could be analyzed using gene ontology to find common disease pathways among transcripts. This study evaluates abnormally regulated transcripts while maintaining spatial resolution, identifies abnormally regulated genes that may reveal previously unknown pathways in ALS pathogenesis, and may ultimately inform therapeutic target selection and biomarker development in this field.

A study used the spatial transcriptomics approach LCM-seq to analyze substantia nigra pars compacta (SNpc) and ventral tegmental area (VTA) dopamine neurons isolated individually from the postmortem brains of 18 individuals (Aguila et al., 2021). The re-identification of 33 markers that are consistently and differentially expressed in SNpc and VTA dopamine neurons, two types of dopamine neurons (DA) that are associated with Parkinson’s disease (PD) and other degenerative diseases, provides important targets for adjusting neuronal vulnerability or analyzing disease. In an additional study (Kamath et al., 2022), they used single nucleus mRNA sequencing to identify 10 transcriptionally distinct subpopulations in an effort to comprehend all cell types in the substantia nigra densa. Slide-seq, a high-resolution spatial transcriptomics method, was also used to locate these populations along the SNpc’s dorsoventral axis. It was discovered that the DA subpopulation SOX6_AGTR1 is extremely sensitive to PD neurodegeneration. Further enrichment analysis revealed that this subpopulation of PD genetic risk genes expresses motifs that may intrinsically affect neurodegeneration in cells. This study reveals the molecular characteristics of DA neurons’ vulnerability and the molecular cascade that leads to their death, which may offer new insights for the improvement of PD experimental models and the development of cell-type-specific or disease-modifying therapies.

To better comprehend the disease, a comprehensive gene expression profile at an early stage is helpful in defining the cellular layers of the brain on a molecular level. As a result, two brain regions associated with the pathogenesis of Alzheimer’s disease (AD) were the focus of a study (Navarro et al., 2020), the hippocampus and olfactory bulb (OFB), by utilizing spatial transcriptomics (ST) to quantify genes that were differentially expressed in the hippocampal and OFB regions of mouse AD models and controls. Additionally, the researchers discovered that hippocampal CA3 expression of BOK genes that are associated with mitochondrial physiology and cell death was decreased in AD patients and mice. It indicating that spatially restricted differential expression of BOK plays a role in the pathology of AD. This study provides a rich resource of spatially differentially expressed genes that will be of great help in understanding the pathology of AD. β-amyloid deposition-induced neuroinflammatory plaques are a common AD pathological change (Xu et al., 2023). A study used spatial transcriptomics (ST) to examine the transcriptional changes occurring in the structural domains of the tissue surrounding amyloid plaques in the APP^NL−G-F^ mouse model to investigate the connection between this pathological change and neurodegeneration (Chen et al., 2020). Early in AD, gene co-expression networks enriched in myelin and oligodendrocyte genes (OLIGs) were found to be altered. It was hypothesized that upregulation of OLIGs might have a protective effect; however, this hypothesis ultimately fell apart as the amyloid plaque load increased. On the other hand, the multicellular gene co-expression network of plaque-induced genes involving the complement system, oxidative stress, lysosomes, and inflammation was particularly prominent in the later stages of the disease. This suggests that complement is an essential component of cellular interactions in the microenvironment of amyloid plaque. Spatial transcriptomic analysis is a novel method for deciphering dysregulated cellular networks near AD and other brain diseases’ pathogenic markers. Plaque-inducible gene’s expression and plaque distance were found to be linked in a recent study (Wood et al., 2022), AD plaque deposition significantly increased the expression of TREM2 and related genes, but not in areas immediately adjacent to plaques. In addition to microglia contacting plaques, TREM2 genotype was highly dependent on this tight regional expression increase. The existence of interactions between plaque and microglia genes is confirmed by this genotype-dependent dependence on plaque contact. In addition, genes differentially expressed in regions associated with pathological changes in AD have been studied using spatial transcriptomics, which may contribute to regional vulnerability in early AD (Chen et al., 2022a). For example, many AD-related signals, such as plaque-induced genes, disease-associated microglia genes, oligodendrocyte-responsive genes, A1 astrocyte genes, and tangle-associated genes, have been identified by ST in the middle temporal gyrus (MTG) 2/3 cortical layer, where excitatory neurons are particularly susceptible to degeneration in early AD. When used in conjunction with smFISH, the genes SLC1A3, KIF5A, SNCG, STMN2, CSRP1, PLP1, Glul, PAQR6, CD9, C1QB, SPP1, CD63, CryAB, and YWHAH were also found to be associated with two major AD pathological markers in layers II/III and V (Aβ plaques and NFTs or neuropil threads). For the purpose of elucidating the pathogenesis of AD and developing disease-modifying therapies for its prevention and treatment, spatial transcriptomics can identify main differences in the disease. Recent spatial transcriptomic studies focusing on inositol polyphosphate-5-phosphatase D (INPP5D) (Castranio et al., 2022), a risk gene for AD, found that knocking down INPP5D in PSAPP mice significantly altered the plaque-specific gene expression profile, exacerbated plaque deposition, and increased the number of microglia associated with plaque. Additionally, CST7 was discovered to be a potentially highly specific marker of AD brain plaques thanks to the presence of a plaque-associated differentially expressed genes signature in spatial transcriptomic analysis. In contrast, another study using an INPP5D-haploinsufficient mouse model (Lin et al., 2022) revealed that INPP5D haploinsufficiency altered pathways related to protein digestion, synaptic plasticity, calcium signaling, and cytokine production in plaque-associated microglia, according to spatial transcriptional profiling of amyloid plaque-associated microglia. It is proposed that INPP5D haploinsufficiency slows neuronal dysfunction and cognitive decline by increasing protective responses in microglia, thereby limiting pathological changes in Aβ. As a result, blocking INPP5D might be an option for AD treatment.

**Neuropsychiatric disorders**: The spatial organization of the brain is fundamentally linked to its function. Based on cell type and density, the neocortex can be divided into six layers, and cells in different layers exhibit distinct patterns of gene expression (Jiang et al., 2015). Localizing spatial gene expression in the human brain at cellular resolution will be essential for advancing our understanding of disease mechanisms because the pathology and gene expression differences that are associated with neuropsychiatric disorders are restricted to particular cortical layers (Kita et al., 2021). To this end, a study sought to identify a spatial gene expression profile in the dorsolateral prefrontal cortex (DLPFC), a brain region associated with numerous neuropsychiatric disorders (Maynard et al., 2021). Several DLPFC laminar flow marker genes were detected, including AQP4 (L1), HPCAL1 (L2), FREM3 (L3), TRABD2A (L5), and KRT17 (L6). In addition, the laminar enrichment of genetic variant genes associated with major depressive disorder (MDD), bipolar disorder (BPD), schizophrenia (SCZD), and autism (ASD) was examined. ST analysis of gene expression in the human DLPFC’s intact spatial organization reveals a broad laminar expression profile, shedding light on crucial functions related to the spatial and molecular definition of cell populations across cortical layers. Furthermore, the hippocampus is one of the brain regions closely associated with the pathogenesis of schizophrenia (Zierhut et al., 2013; Lieberman et al., 2018). Using LCM-seq technology, a study examined the human hippocampal dentate gyrus granular cell layer (DG-GCL) for cell type enrichment expression analysis (Jaffe et al., 2020). Among the 9 million expressed quantitative trait loci (eQTLs) identified in the DG-GCL, expression of 15 transcriptional signatures associated with schizophrenia risk loci were identified, including PSD3, MARS, NLGN4X, GRM3, SEMA6D, MMP16, THEMIS, SATB2, CACNA1C, KCTD18, PRKD1, HDAC2-AS2. GRM3 and CACNA1c, which respectively encode G protein-coupled receptors and ion channels, are typical targets for schizophrenia drug therapy in this context. The development of experimental models for the treatment of psychiatric disorders can greatly benefit from the discovery of these cell type-specific associations.

**Stroke:** The heme released after intracranial hemorrhage either generates superoxide or interactions with cells to cause an inflammatory response, leading to secondary brain damage (Chen et al., 2022b). To validate the pro-inflammatory activity of heme and characterize its key phenotypes causing secondary brain injury, a study injected different doses of heme into a mouse model and performed spatial transcriptome analysis to define the brain heme response signature (Buzzi et al., 2022). ST data also showed that heme activates inflammation through multiple synergistic signaling cascades. It leads to marked dose-dependent inflammation, as well as localized disruption of BBB function, brain edema, perfusion deficit and severe neurological impairment. Hemopexin (Hpx), on the other hand, reduces the effects of cerebral heme on gene expression, radiological abnormalities, and neurological deficits. As a result, Hpx supplementation might be an option for treating intracranial hemorrhage (Akeret et al., 2022; Qiu et al., 2022).

**Epilepsy**: Neuroinflammation may exacerbate seizures, according to clinical and experimental research (Soltani Khaboushan et al., 2022). During the early stages of epileptic activity, immune cell activation releases cytokines that alter epileptic thresholds by triggering transcriptional signals in glial cells or the microenvironment (Iori et al., 2016). Using 10x Genomics spatial transcriptomics, a recent study used models of epilepsy to observe transcriptional changes in specific brain regions in mice (Zhang et al., 2022b). A distinct regional transcriptome signature was found in the hippocampus of epileptic mice. Moreover, the hippocampus exhibited a unique inflammatory gene profile, including glial cell activation, apoptosis and immune response. C-C chemokine ligand 5 (CCL5) was found to be significantly expressed among all differentially expressed genes. However, in epileptic mice, Maraviroc, a C-C chemokine receptor 5(CCR5) antagonist, prevented microglia activation and neuronal degeneration, thereby reducing the activity of epilepsy. Neuroinflammation after seizures may be targeted by CCL5/CCR5 signaling, as suggested by the outcome. This study provides new insights into immune interventions for seizure activity, maps brain region-specific gene expression profiles, and enhances our understanding of the inflammatory profile of seizures.

There is a high prevalence of memory impairment in patients with temporal lobe epilepsy (TLE) (Busch et al., 2022b). Using the novel method called spatial transcriptomics, the molecular changes associated with TLE memory impairment can now be seen, and biomarkers of the disease can be found (Busch et al., 2022a). The differential expression of transcripts in four brain regions (the dentate gyrus, CA3, CA1, and neocortex) associated with episodic memory in TLE patients was spatially quantified in a recent study using spatial transcriptomic techniques. Numerous differentially expressed transcripts (DETs) were found in the hippocampus and neocortex, two memory subregions, according to the findings. DETs in the hippocampal subregions involve genes related to neuritogenesis and long-term potential, processes that are essential for the formation of new memories. Numerous DETs in the neocortex are associated with neurodegenerative diseases. The hippocampal CA3 subregions show the strongest molecular signature of the temporal lobe subregions’ distinct roles in the molecular changes associated with memory impairment. BDNF was found to be the center of CA3-related networks that control phenotype-related processes like cognition, memory, long-term potentiation, and neurocytogenesis after analysis.

Burst discharges not only appear in animal models of brain tumors, but also in the peritumoral area of patients with malignant brain tumors during biopsy. In patients with malignant brain tumors, burst discharges in the peritumoral region may be a source of epileptic activity. In addition, growing evidence suggests that interactions between gliomas and neurons in the peritumoral region are necessary for the occurrence of epileptic discharges. A recent study established a rat glioma model and characterized it at the cellular and molecular levels in order to investigate the biological changes associated with the peritumoral region (Komiyama et al., 2022).

A transcriptome analysis using LCM-seq in brain tissue sections of a rat glioma model identified 19 genes that were differentially expressed in the peritumoral region (Komiyama et al., 2022). Five of these genes (GFAP, GMPPA, TUBB2B, SLC22A8, and PLXNB3) were linked to epilepsy or neurodevelopmental disorders. In addition, 31 typical signaling pathways that could be actively altered in the peritumoral region were predicted by this study. These findings contribute to a deeper comprehension of the pathophysiological mechanisms of glioma-associated epilepsy and suggest that biological changes in the peritumoral region may be the cause of the condition Supplementary Table S1.

## Conclusion and future directions

Spatial transcriptomic technologies provide new insights to explore the molecular mechanisms of disease from the perspective of spatial heterogeneity and offer new means to discover potential biomarkers and predict disease progression, playing a huge application within the diagnosis and treatment of neurological diseases. Despite the expansion of spatial transcriptomic innovations, there is currently no single spatial transcriptomic technique suitable for all situations. These spatial transcriptomic methods still need to be enhanced for experimental studies. In general, these technologies have the following defects that need to be improved: 1) High-resolution profiles can be obtained using microdissection-based techniques, but they include laborious processes and are difficult to use. 2) Target mRNA can be detected using in situ hybridization techniques, but smFISH is limited by the amount of fluorophores, seqFISH, and MERFISH are expensive studies, and probe hybridization can be mistaken. 3) Technologies using in situ sequencing have a low detection throughput and can only perform targeted detection. 4) While increasing resolution and throughput, in situ capture technologies can only reach the level of a single cell. (4) Live cell-based spatial transcriptome technology is not entirely suitable to the study of human materials. In the Supplementary Material section, a summary table with specifics on sample type, approach, spatial resolution, throughput, detection level (depth), advantages and restrictions could well be noticed.

Over all, future spatial transcriptome technologies must therefore be improved and enhanced. In order to fully describe and comprehend the microenvironment of brain tissue, the spatial transcriptome can combine with other spatial histology technologies like spatial proteomics to discover protein expression and localization patterns at the single-cell level. This will make it easier to show a complete and accurate brain cell atlas as anticipated from the Brain Initiative Cell Census Network (BICCN) and the Human Cell Atlas. In addition, multi-omics synthesis provides reliable information for investigating disease causation, finding therapeutic targets, and discovering new biomarkers. Secondly, combining spatial transcriptomic data with multimodal data, such as calcium imaging and/or optogenetic probing, can also produce interpretations of circuit activity that are specific to particular cell types. This can take full advantage of the spatial transcriptome to unravel the complex intercellular interactions, gene regulatory networks and subcellular structures under physiological and pathological conditions.

## References

[B1] Aguila J. Cheng S. Kee N. Cao M. Wang M. Deng Q. (2021). Spatial RNA sequencing identifies robust markers of vulnerable and resistant human midbrain dopamine neurons and their expression in Parkinson’s disease. Front. Mol. Neurosci. 14, 699562. 10.3389/fnmol.2021.699562 34305528 PMC8297217

[B2] Aguilar-Bravo B. Sancho-Bru P. (2019). Laser capture microdissection: Techniques and applications in liver diseases. Hepatol. Int. 13 (2), 138–147. 10.1007/s12072-018-9917-3 30600479

[B3] Akeret K. Hugelshofer M. Schaer D. J. Buzzi R. M. (2022). Spatial transcriptome data from coronal mouse brain sections after striatal injection of heme and heme-hemopexin. Data Brief. 41, 107866. 10.1016/j.dib.2022.107866 35141374 PMC8814302

[B4] Almeida D. Turecki G. (2022). Profiling cell-type specific gene expression in post-mortem human brain samples through laser capture microdissection. Methods 207, 3–10. 10.1016/j.ymeth.2022.08.013 36064002

[B5] Asp M. Bergenstrahle J. Lundeberg J. (2020). Spatially resolved transcriptomes-next generation tools for tissue exploration. Bioessays 42 (10), e1900221. 10.1002/bies.201900221 32363691

[B6] Bao F. Deng Y. Wan S. Shen S. Q. Wang B. Dai Q. (2022). Integrative spatial analysis of cell morphologies and transcriptional states with MUSE. Nat. Biotechnol. 40 (8), 1200–1209. 10.1038/s41587-022-01251-z 35347329

[B7] Bhamidipati T. Sinha M. Sen C. K. Singh K. (2022). Laser capture microdissection in the spatial analysis of epigenetic modifications in skin: A comprehensive review. Oxid. Med. Cell Longev. 2022, 4127238. 10.1155/2022/4127238 35186184 PMC8850045

[B8] Biancalani T. Scalia G. Buffoni L. Avasthi R. Lu Z. Sanger A. (2021). Deep learning and alignment of spatially resolved single-cell transcriptomes with Tangram. Nat. Methods 18 (11), 1352–1362. 10.1038/s41592-021-01264-7 34711971 PMC8566243

[B9] Boogerd C. J. Lacraz G. P. A. Vertesy A. Van Kampen S. J. Perini I. De Ruiter H. (2022). Spatial transcriptomics unveils ZBTB11 as a regulator of cardiomyocyte degeneration in arrhythmogenic cardiomyopathy. Cardiovasc Res. 2022, cvac072. 10.1093/cvr/cvac072 PMC10064846 35576477

[B10] Busch R. M. Yehia L. Blumcke I. Hu B. Prayson R. Hermann B. P. (2022a). Molecular and subregion mechanisms of episodic memory phenotypes in temporal lobe epilepsy. Brain Commun. 4 (6), fcac285. 10.1093/braincomms/fcac285 36419965 PMC9679425

[B11] Busch R. M. Yehia L. Hu B. Goldman M. Hermann B. P. Najm I. M. (2022b). Brain single cell transcriptomic profiles in episodic memory phenotypes associated with temporal lobe epilepsy. NPJ Genom Med. 7 (1), 69. 10.1038/s41525-022-00339-4 36446800 PMC9709106

[B12] Buzzi R. M. Akeret K. Schwendinger N. Klohs J. Vallelian F. Hugelshofer M. (2022). Spatial transcriptome analysis defines heme as a hemopexin-targetable inflammatoxin in the brain. Free Radic. Biol. Med. 179, 277–287. 10.1016/j.freeradbiomed.2021.11.011 34793930

[B13] Casasent A. K. Schalck A. Gao R. Sei E. Long A. Pangburn W. (2018). Multiclonal invasion in breast tumors identified by topographic single cell sequencing. Cell 172 (1-2), 205–217. 10.1016/j.cell.2017.12.007 29307488 PMC5766405

[B14] Castranio E. L. Hasel P. Haure-Mirande J. V. Ramirez Jimenez A. V. Hamilton B. W. Kim R. D. (2022). Microglial INPP5D limits plaque formation and glial reactivity in the PSAPP mouse model of Alzheimer's disease. Alzheimers Dement., 1–14. 10.1002/alz.12821 PMC10481344 36448627

[B15] Chang C. C. Chong H. T. Tashiro A. (2021). Laser capture microdissection of single neurons with morphological visualization using fluorescent proteins fused to transmembrane proteins. eNeuro 8, 0275. 10.1523/ENEURO.0275-20.2021 PMC8422851 34400471

[B16] Chen H. Murray E. Sinha A. Laumas A. Li J. Lesman D. (2021). Dissecting mammalian spermatogenesis using spatial transcriptomics. Cell Rep. 37 (5), 109915. 10.1016/j.celrep.2021.109915 34731600 PMC8606188

[B17] Chen J. Suo S. Tam P. P. Han J. J. PengandJING G. N. (2017). Spatial transcriptomic analysis of cryosectioned tissue samples with Geo-seq. Nat. Protoc. 12 (3), 566–580. 10.1038/nprot.2017.003 28207000

[B18] Chen K. H. Boettiger A. N. Moffitt J. R. WangandZHUANG S. X. (2015). RNA imaging. Spatially resolved, highly multiplexed RNA profiling in single cells. Science 348 (6233), aaa6090. 10.1126/science.aaa6090 25858977 PMC4662681

[B19] Chen S. Chang Y. Li L. Acosta D. Li Y. Guo Q. (2022a). Spatially resolved transcriptomics reveals genes associated with the vulnerability of middle temporal gyrus in Alzheimer's disease. Acta Neuropathol. Commun. 10 (1), 188. 10.1186/s40478-022-01494-6 36544231 PMC9773466

[B20] Chen S. Li L. Peng C. Bian C. Ocak P. E. Zhang J. H. (2022b). Targeting oxidative stress and inflammatory response for blood-brain barrier protection in intracerebral hemorrhage. Antioxid. Redox Signal 37 (1-3), 115–134. 10.1089/ars.2021.0072 35383484

[B21] Chen W. T. Lu A. Craessaerts K. Pavie B. Sala Frigerio C. Corthout N. (2020). Spatial transcriptomics and *in situ* sequencing to study Alzheimer's disease. Cell 182 (4), 976–991. 10.1016/j.cell.2020.06.038 32702314

[B22] Chen X. Sun Y. C. Church G. M. LeeandZADOR J. H. A. M. (2018). Efficient *in situ* barcode sequencing using padlock probe-based BaristaSeq. Nucleic Acids Res. 46 (4), e22. 10.1093/nar/gkx1206 29190363 PMC5829746

[B23] Chen X. Sun Y. C. Zhan H. Kebschull J. M. Fischer S. Matho K. (2019). High-throughput mapping of long-range neuronal projection using *in situ* sequencing. Cell 179 (3), 772–786. 10.1016/j.cell.2019.09.023 31626774 PMC7836778

[B24] Cho C. S. XI J. Si Y. Park S. R. Hsu J. E. Kim M. (2021). Microscopic examination of spatial transcriptome using Seq-Scope. Cell 184 (13), 3559–3572.e22. 10.1016/j.cell.2021.05.010 34115981 PMC8238917

[B25] Civita P. Franceschi S. Aretini P. Ortenzi V. Menicagli M. Lessi F. (2019). Laser capture microdissection and RNA-seq analysis: High sensitivity approaches to explain histopathological heterogeneity in human glioblastoma FFPE archived tissues. Front. Oncol. 9, 482. 10.3389/fonc.2019.00482 31231613 PMC6568189

[B26] Do T. H. Ma F. Andrade P. R. Teles R. De Andrade Silva B. J. Hu C. (2022). TREM2 macrophages induced by human lipids drive inflammation in acne lesions. Sci. Immunol. 7 (73), eabo2787. 10.1126/sciimmunol.abo2787 35867799 PMC9400695

[B27] Eng C. L. Lawson M. Zhu Q. Dries R. Koulena N. Takei Y. (2019). Transcriptome-scale super-resolved imaging in tissues by RNA seqFISH. Nature 568 (7751), 235–239. 10.1038/s41586-019-1049-y 30911168 PMC6544023

[B28] Fang R. Xia C. Close J. L. Zhang M. He J. Huang Z. (2022). Conservation and divergence of cortical cell organization in human and mouse revealed by MERFISH. Science 377 (6601), 56–62. 10.1126/science.abm1741 35771910 PMC9262715

[B29] Fazal F. M. Han S. Parker K. R. Kaewsapsak P. Xu J. Boettiger A. N. (2019). Atlas of subcellular RNA localization revealed by APEX-seq. Cell 178 (2), 473–490. 10.1016/j.cell.2019.05.027 31230715 PMC6786773

[B30] Goh J. J. L. Chou N. Seow W. Y. Ha N. Cheng C. P. P. Chang Y. C. (2020). Highly specific multiplexed RNA imaging in tissues with split-FISH. Nat. Methods 17 (7), 689–693. 10.1038/s41592-020-0858-0 32541852

[B31] Gregory J. M. Mcdade K. Livesey M. R. Croy I. Marion De Proce S. Aitman T. (2020). Spatial transcriptomics identifies spatially dysregulated expression of GRM3 and USP47 in amyotrophic lateral sclerosis. Neuropathol. Appl. Neurobiol. 46 (5), 441–457. 10.1111/nan.12597 31925813

[B32] Griesser E. Wyatt H. Ten Have S. Stierstorfer B. LenterandLAMOND M. A. I. (2020). Quantitative profiling of the human substantia nigra proteome from laser-capture microdissected FFPE tissue. Mol. Cell Proteomics 19 (5), 839–851. 10.1074/mcp.RA119.001889 32132230 PMC7196589

[B33] Haimovich G. Gerst J. E. (2018). Single-molecule fluorescence *in situ* hybridization (smFISH) for RNA detection in adherent animal cells. Bio Protoc. 8 (21), e3070. 10.21769/BioProtoc.3070 PMC8342053 34532531

[B34] Hashimshony T. Senderovich N. Avital G. Klochendler A. De Leeuw Y. Anavy L. (2016). CEL-Seq2: Sensitive highly-multiplexed single-cell RNA-seq. Genome Biol. 17, 77. 10.1186/s13059-016-0938-8 27121950 PMC4848782

[B35] Holler K. Junker J. P. (2019). RNA tomography for spatially resolved transcriptomics (Tomo-Seq). Methods Mol. Biol. 1920, 129–141. 10.1007/978-1-4939-9009-2_9 30737690

[B36] Hu K. H. Eichorst J. P. Mcginnis C. S. Patterson D. M. Chow E. D. Kersten K. (2020). ZipSeq: Barcoding for real-time mapping of single cell transcriptomes. Nat. Methods 17 (8), 833–843. 10.1038/s41592-020-0880-2 32632238 PMC7891292

[B37] Iegorova V. Naraine R. Psenicka M. ZelazowskaandSINDELKA M. R. (2022). Comparison of RNA localization during oogenesis within *Acipenser ruthenus* and *Xenopus laevis* . Front. Cell Dev. Biol. 10, 982732. 10.3389/fcell.2022.982732 36204678 PMC9531136

[B38] Imbert A. Ouyang W. Safieddine A. Coleno E. Zimmer C. Bertrand E. (2022). FISH-Quant v2: A scalable and modular tool for smFISH image analysis. RNA 28 (6), 786–795. 10.1261/rna.079073.121 35347070 PMC9074904

[B39] Iori V. Frigerio F. Vezzani F. A. (2016). Modulation of neuronal excitability by immune mediators in epilepsy. Curr. Opin. Pharmacol. 26, 118–123. 10.1016/j.coph.2015.11.002 26629681 PMC4716878

[B40] Jaffe A. E. Hoeppner D. J. Saito T. Blanpain L. Ukaigwe J. Burke E. E. (2020). Profiling gene expression in the human dentate gyrus granule cell layer reveals insights into schizophrenia and its genetic risk. Nat. Neurosci. 23 (4), 510–519. 10.1038/s41593-020-0604-z 32203495

[B41] Jiang X. Shen S. Cadwell C. R. Berens P. Sinz F. Ecker A. S. (2015). Principles of connectivity among morphologically defined cell types in adult neocortex. Science 350 (6264), aac9462. 10.1126/science.aac9462 26612957 PMC4809866

[B42] Kamath T. Abdulraouf A. Burris S. J. Langlieb J. Gazestani V. Nadaf N. M. (2022). Single-cell genomic profiling of human dopamine neurons identifies a population that selectively degenerates in Parkinson's disease. Nat. Neurosci. 25 (5), 588–595. 10.1038/s41593-022-01061-1 35513515 PMC9076534

[B43] Kaufmann M. Schaupp A. L. Sun R. Coscia F. Dendrou C. A. Cortes A. (2022). Identification of early neurodegenerative pathways in progressive multiple sclerosis. Nat. Neurosci. 25 (7), 944–955. 10.1038/s41593-022-01097-3 35726057

[B44] Kebschull J. M. Richman E. B. Ringach N. Friedmann D. Albarran E. Kolluru S. S. (2020). Cerebellar nuclei evolved by repeatedly duplicating a conserved cell-type set. Science 370 (6523), eabd5059. 10.1126/science.abd5059 33335034 PMC8510508

[B45] Kim D. W. Yao Z. Graybuck L. T. Kim T. K. Nguyen T. N. Smith K. A. (2019). Multimodal analysis of cell types in a hypothalamic node controlling social behavior. Cell 179 (3), 713–728. 10.1016/j.cell.2019.09.020 31626771 PMC7534821

[B46] Kishi J. Y. Liu N. West E. R. Sheng K. Jordanides J. J. Serrata M. (2022). Light-seq: Light-directed *in situ* barcoding of biomolecules in fixed cells and tissues for spatially indexed sequencing. Nat. Methods 19 (11), 1393–1402. 10.1038/s41592-022-01604-1 36216958 PMC9636025

[B47] Kita Y. Nishibe H. Wang Y. Hashikawa T. Kikuchi S. S. U M. (2021). Cellular-resolution gene expression profiling in the neonatal marmoset brain reveals dynamic species- and region-specific differences. Proc. Natl. Acad. Sci. U. S. A. 118 (18), e2020125118. 10.1073/pnas.2020125118 33903237 PMC8106353

[B48] Kleino I. Frolovaite P. SuomiandELO T. L. L. (2022). Computational solutions for spatial transcriptomics. Comput. Struct. Biotechnol. J. 20, 4870–4884. 10.1016/j.csbj.2022.08.043 36147664 PMC9464853

[B49] Kocks C. Boltengagen A. Piwecka M. Rybak-Wolf A. Rajewsky N. (2018). Single-molecule fluorescence *in situ* hybridization (FISH) of circular RNA CDR1as. Methods Mol. Biol. 1724, 77–96. 10.1007/978-1-4939-7562-4_7 29322442

[B50] Komiyama K. Iijima K. Kawabata-Iwakawa R. Fujihara K. Kakizaki T. Yanagawa Y. (2022). Glioma facilitates the epileptic and tumor-suppressive gene expressions in the surrounding region. Sci. Rep. 12 (1), 6805. 10.1038/s41598-022-10753-4 35474103 PMC9042955

[B51] Kruse F. Junker J. P. Van Oudenaarden A. Bakkers J. (2016). Tomo-seq: A method to obtain genome-wide expression data with spatial resolution. Methods Cell Biol. 135, 299–307. 10.1016/bs.mcb.2016.01.006 27443932

[B52] Kwon S. (2013). Single-molecule fluorescence *in situ* hybridization: Quantitative imaging of single RNA molecules. BMB Rep. 46 (2), 65–72. 10.5483/bmbrep.2013.46.2.016 23433107 PMC4133856

[B53] Lacraz G. P. A. Junker J. P. Gladka M. M. Molenaar B. Scholman K. T. Vigil-Garcia M. (2017). Tomo-seq identifies SOX9 as a key regulator of cardiac fibrosis during ischemic injury. Circulation 136 (15), 1396–1409. 10.1161/CIRCULATIONAHA.117.027832 28724751

[B54] Lahnemann D. Koster J. Szczurek E. Mccarthy D. J. Hicks S. C. Robinson M. D. (2020). Eleven grand challenges in single-cell data science. Genome Biol. 21 (1), 31. 10.1186/s13059-020-1926-6 32033589 PMC7007675

[B55] Larsson L. Frisen J. Lundeberg J. (2021). Spatially resolved transcriptomics adds a new dimension to genomics. Nat. Methods 18 (1), 15–18. 10.1038/s41592-020-01038-7 33408402

[B56] Lee J. H. Daugharthy E. R. Scheiman J. Kalhor R. Ferrante T. C. Terry R. (2015). Fluorescent *in situ* sequencing (FISSEQ) of RNA for gene expression profiling in intact cells and tissues. Nat. Protoc. 10 (3), 442–458. 10.1038/nprot.2014.191 25675209 PMC4327781

[B57] Lee J. H. Daugharthy E. R. Scheiman J. Kalhor R. Yang J. L. Ferrante T. C. (2014). Highly multiplexed subcellular RNA sequencing *in situ* . Science 343 (6177), 1360–1363. 10.1126/science.1250212 24578530 PMC4140943

[B58] Lee J. H. (2017). Quantitative approaches for investigating the spatial context of gene expression. Wiley Interdiscip. Rev. Syst. Biol. Med. 9 (2), e1369. 10.1002/wsbm.1369 28001340 PMC5315614

[B59] Lepine S. Castellanos-Montiel M. J. Durcan T. M. (2022). TDP-43 dysregulation and neuromuscular junction disruption in amyotrophic lateral sclerosis. Transl. Neurodegener. 11 (1), 56. 10.1186/s40035-022-00331-z 36575535 PMC9793560

[B60] Lewis S. M. Asselin-Labat M. L. Nguyen Q. Berthelet J. Tan X. Wimmer V. C. (2021). Spatial omics and multiplexed imaging to explore cancer biology. Nat. Methods 18 (9), 997–1012. 10.1038/s41592-021-01203-6 34341583

[B61] Li R. Zou Z. Wang W. Zou P. (2022). Metabolic incorporation of electron-rich ribonucleosides enhances APEX-seq for profiling spatially restricted nascent transcriptome. Cell Chem. Biol. 29 (7), 1218–1231. 10.1016/j.chembiol.2022.02.005 35245437

[B62] Liao J. Lu X. Shao X. Zhu L. Fan X. (2021). Uncovering an organ's molecular architecture at single-cell resolution by spatially resolved transcriptomics. Trends Biotechnol. 39 (1), 43–58. 10.1016/j.tibtech.2020.05.006 32505359

[B63] Lieberman J. A. Girgis R. R. Brucato G. Moore H. Provenzano F. Kegeles L. (2018). Hippocampal dysfunction in the pathophysiology of schizophrenia: A selective review and hypothesis for early detection and intervention. Mol. Psychiatry 23 (8), 1764–1772. 10.1038/mp.2017.249 29311665 PMC6037569

[B64] Lin P. B. Tsai A. P. Soni D. Lee-Gosselin A. Moutinho M. Puntambekar S. S. (2022). INPP5D deficiency attenuates amyloid pathology in a mouse model of Alzheimer's disease. Alzheimers Dement., 1–10. 10.1002/alz.12849 36524682

[B65] Liu J. Tran V. Vemuri V. N. P. Byrne A. Borja M. Kim Y. J. (2023). Concordance of MERFISH spatial transcriptomics with bulk and single-cell RNA sequencing. Life Sci. Alliance 6 (1), e202201701. 10.26508/lsa.202201701 36526371 PMC9760489

[B66] Liu S. Punthambaker S. Iyer E. P. R. Ferrante T. Goodwin D. Furth D. (2021). Barcoded oligonucleotides ligated on RNA amplified for multiplexed and parallel *in situ* analyses. Nucleic Acids Res. 49 (10), e58. 10.1093/nar/gkab120 33693773 PMC8191787

[B67] Liu X. Jiang Y. Song D. Zhang L. Xu G. Hou R. (2022). Clinical challenges of tissue preparation for spatial transcriptome. Clin. Transl. Med. 12 (1), e669. 10.1002/ctm2.669 35083877 PMC8792118

[B68] Liu Y. Yang M. Deng Y. Su G. Enninful A. Guo C. C. (2020). High-spatial-resolution multi-omics sequencing via deterministic barcoding in tissue. Cell 183 (6), 1665–1681. 10.1016/j.cell.2020.10.026 33188776 PMC7736559

[B121] Lohoff T. Ghazanfar S. Missarova A. Koulena N. Pierson N. Griffiths J. A. (2022). Integration of spatial and single-cell transcriptomic data elucidates mouse organogenesis. Nat. Biotechnol. 40 (1), 74–85. 10.1038/s41587-021-01006-2 34489600 PMC8763645

[B69] Lovatt D. Ruble B. K. Lee J. Dueck H. Kim T. K. Fisher S. (2014). Transcriptome *in vivo* analysis (TIVA) of spatially defined single cells in live tissue. Nat. Methods 11 (2), 190–196. 10.1038/nmeth.2804 24412976 PMC3964595

[B70] Lu Y. Liu M. Yang J. Weissman S. M. Pan X. Katz S. G. (2021). Spatial transcriptome profiling by MERFISH reveals fetal liver hematopoietic stem cell niche architecture. Cell Discov. 7 (1), 47. 10.1038/s41421-021-00266-1 34183665 PMC8238952

[B71] Lubeck E. Coskun A. F. Zhiyentayev T. Ahmad M. Cai L. (2014). Single-cell *in situ* RNA profiling by sequential hybridization. Nat. Methods 11 (4), 360–361. 10.1038/nmeth.2892 24681720 PMC4085791

[B72] Maniatis S. Aijo T. Vickovic S. Braine C. Kang K. Mollbrink A. (2019). Spatiotemporal dynamics of molecular pathology in amyotrophic lateral sclerosis. Science 364 (6435), 89–93. 10.1126/science.aav9776 30948552

[B73] Maynard K. R. Collado-Torres L. Weber L. M. Uytingco C. Barry B. K. Williams S. R. (2021). Transcriptome-scale spatial gene expression in the human dorsolateral prefrontal cortex. Nat. Neurosci. 24 (3), 425–436. 10.1038/s41593-020-00787-0 33558695 PMC8095368

[B74] Miller B. F. Huang F. Atta L. Sahoo A. Fan J. (2022). Reference-free cell type deconvolution of multi-cellular pixel-resolution spatially resolved transcriptomics data. Nat. Commun. 13 (1), 2339. 10.1038/s41467-022-30033-z 35487922 PMC9055051

[B75] Misrielal C. Alsema A. M. Wijering M. H. C. Miedema A. Mauthe M. Reggiori F. (2022). Transcriptomic changes in autophagy-related genes are inversely correlated with inflammation and are associated with multiple sclerosis lesion pathology. Brain Behav. Immun. Health 25, 100510. 10.1016/j.bbih.2022.100510 36120103 PMC9478930

[B76] Moffitt J. R. Lundberg E. Heyn H. (2022). The emerging landscape of spatial profiling technologies. Nat. Rev. Genet. 23 (12), 741–759. 10.1038/s41576-022-00515-3 35859028

[B77] Moffitt J. R. Zhuang X. (2016). RNA imaging with multiplexed error-robust fluorescence *in situ* hybridization (MERFISH). Methods Enzymol. 572, 1–49. 10.1016/bs.mie.2016.03.020 27241748 PMC5023431

[B78] Moncada R. Barkley D. Wagner F. Chiodin M. Devlin J. C. Baron M. (2020). Integrating microarray-based spatial transcriptomics and single-cell RNA-seq reveals tissue architecture in pancreatic ductal adenocarcinomas. Nat. Biotechnol. 38 (3), 333–342. 10.1038/s41587-019-0392-8 31932730

[B79] Moses L. Pachter L. (2022). Museum of spatial transcriptomics. Nat. Methods 19 (5), 534–546. 10.1038/s41592-022-01409-2 35273392

[B80] Naraine R. Iegorova V. Abaffy P. Franek R. Soukup V. Psenicka M. (2022). Evolutionary conservation of maternal RNA localization in fishes and amphibians revealed by TOMO-Seq. Dev. Biol. 489, 146–160. 10.1016/j.ydbio.2022.06.013 35752299

[B81] Navarro J. F. Croteau D. L. Jurek A. Andrusivova Z. Yang B. Wang Y. (2020). Spatial transcriptomics reveals genes associated with dysregulated mitochondrial functions and stress signaling in alzheimer disease. iScience 23 (10), 101556. 10.1016/j.isci.2020.101556 33083725 PMC7522123

[B82] Nguyen H. Q. Chattoraj S. Castillo D. Nguyen S. C. Nir G. Lioutas A. (2020). 3D mapping and accelerated super-resolution imaging of the human genome using *in situ* sequencing. Nat. Methods 17 (8), 822–832. 10.1038/s41592-020-0890-0 32719531 PMC7537785

[B83] Nichterwitz S. Chen G. Aguila Benitez J. Yilmaz M. Storvall H. Cao M. (2016). Laser capture microscopy coupled with Smart-seq2 for precise spatial transcriptomic profiling. Nat. Commun. 7, 12139. 10.1038/ncomms12139 27387371 PMC4941116

[B84] Padron A. Iwasaki S. Ingolia N. T. (2019). Proximity RNA labeling by APEX-seq reveals the organization of translation initiation complexes and repressive RNA granules. Mol. Cell 75 (4), 875–887. 10.1016/j.molcel.2019.07.030 31442426 PMC6834362

[B85] Paul I. White C. Turcinovic I. Emili A. (2021). Imaging the future: The emerging era of single-cell spatial proteomics. FEBS J. 288 (24), 6990–7001. 10.1111/febs.15685 33351222

[B86] Piskadlo E. Eichenberger B. T. Giorgetti L. Chao J. A. (2022). Design, labeling, and application of probes for RNA smFISH. Methods Mol. Biol. 2537, 173–183. 10.1007/978-1-0716-2521-7_10 35895264

[B87] Qiu Z. Li S. Luo M. Zhu S. Wang Z. Jiang Y. (2022). Detection of differentially expressed genes in spatial transcriptomics data by spatial analysis of spatial transcriptomics: A novel method based on spatial statistics. Front. Neurosci. 16, 1086168. 10.3389/fnins.2022.1086168 36523429 PMC9745188

[B88] Rao A. Barkley D. Franca G. S. Yanai I. (2021). Exploring tissue architecture using spatial transcriptomics. Nature 596 (7871), 211–220. 10.1038/s41586-021-03634-9 34381231 PMC8475179

[B89] Rao B. H. Soucek P. Hlavac V. (2022). Laser capture microdissection: A gear for pancreatic cancer research. Int. J. Mol. Sci. 23 (23), 14566. 10.3390/ijms232314566 36498893 PMC9741023

[B90] Ren X. Kang B. Zhang Z. (2018). Understanding tumor ecosystems by single-cell sequencing: Promises and limitations. Genome Biol. 19 (1), 211. 10.1186/s13059-018-1593-z 30509292 PMC6276232

[B91] Rodriques S. G. Stickels R. R. Goeva A. Martin C. A. Murray E. Vanderburg C. R. (2019). Slide-seq: A scalable technology for measuring genome-wide expression at high spatial resolution. Science 363 (6434), 1463–1467. 10.1126/science.aaw1219 30923225 PMC6927209

[B92] Schneider A. F. L. Hackenberger C. P. R. (2017). Fluorescent labelling in living cells. Curr. Opin. Biotechnol. 48, 61–68. 10.1016/j.copbio.2017.03.012 28395178

[B93] Shah S. Lubeck E. Zhou W. Cai L. (2016). *In situ* transcription profiling of single cells reveals spatial organization of cells in the mouse Hippocampus. Neuron 92 (2), 342–357. 10.1016/j.neuron.2016.10.001 27764670 PMC5087994

[B94] Shah S. Lubeck E. Zhou W. CAI L. (2017). seqFISH accurately detects transcripts in single cells and reveals robust spatial organization in the Hippocampus. Neuron 94 (4), 752–758. 10.1016/j.neuron.2017.05.008 28521130

[B95] Soltani Khaboushan A. Yazdanpanah N. Rezaei N. (2022). Neuroinflammation and proinflammatory cytokines in epileptogenesis. Mol. Neurobiol. 59 (3), 1724–1743. 10.1007/s12035-022-02725-6 35015252

[B96] Srivatsan S. R. Regier M. C. Barkan E. Franks J. M. Packer J. S. Grosjean P. (2021). Embryo-scale, single-cell spatial transcriptomics. Science 373 (6550), 111–117. 10.1126/science.abb9536 34210887 PMC9118175

[B97] Stahl P. L. Salmen F. Vickovic S. Lundmark A. Navarro J. F. Magnusson J. (2016). Visualization and analysis of gene expression in tissue sections by spatial transcriptomics. Science 353 (6294), 78–82. 10.1126/science.aaf2403 27365449

[B98] Stark R. Grzelak M. Hadfield J. (2019). RNA sequencing: The teenage years. Nat. Rev. Genet. 20 (11), 631–656. 10.1038/s41576-019-0150-2 31341269

[B99] Stickels R. R. Murray E. Kumar P. Li J. Marshall J. L. Di Bella D. J. (2021). Highly sensitive spatial transcriptomics at near-cellular resolution with Slide-seqV2. Nat. Biotechnol. 39 (3), 313–319. 10.1038/s41587-020-0739-1 33288904 PMC8606189

[B100] Su G. Qin X. Enninful A. Bai Z. Deng Y. Liu Y. (2021). Spatial multi-omics sequencing for fixed tissue via DBiT-seq. Star. Protoc. 2 (2), 100532. 10.1016/j.xpro.2021.100532 34027489 PMC8132129

[B101] Takei Y. Yun J. Zheng S. Ollikainen N. Pierson N. White J. (2021). Integrated spatial genomics reveals global architecture of single nuclei. Nature 590 (7845), 344–350. 10.1038/s41586-020-03126-2 33505024 PMC7878433

[B102] Takeuchi K. Soda M. Togashi Y. Suzuki R. Sakata S. Hatano S. (2012). RET, ROS1 and ALK fusions in lung cancer. Nat. Med. 18 (3), 378–381. 10.1038/nm.2658 22327623

[B103] Togashi Y. Dobashi A. Sakata S. Sato Y. Baba S. Seto A. (2018). MYB and MYBL1 in adenoid cystic carcinoma: Diversity in the mode of genomic rearrangement and transcripts. Mod. Pathol. 31 (6), 934–946. 10.1038/s41379-018-0008-8 29410490

[B104] Vickovic S. Eraslan G. Salmen F. Klughammer J. Stenbeck L. Schapiro D. (2019). High-definition spatial transcriptomics for *in situ* tissue profiling. Nat. Methods 16 (10), 987–990. 10.1038/s41592-019-0548-y 31501547 PMC6765407

[B105] Wan X. Bryant S. M. Hart G. (2003). A topographical study of mechanical and electrical properties of single myocytes isolated from normal Guinea-pig ventricular muscle. J. Anat. 202 (6), 525–536. 10.1046/j.1469-7580.2003.00187.x 12846474 PMC1571105

[B106] Wang N. Li X. Wang R. DING Z. (2021). Spatial transcriptomics and proteomics technologies for deconvoluting the tumor microenvironment. Biotechnol. J. 16(9), e2100041. 10.1002/biot.202100041 34125481

[B107] Wang S. (2019). Single molecule RNA FISH (smFISH) in whole-mount mouse embryonic organs. Curr. Protoc. Cell Biol. 83 (1), e79. 10.1002/cpcb.79 30394692 PMC6500779

[B108] Wang X. Allen W. E. Wright M. A. Sylwestrak E. L. Samusik N. Vesuna S. (2018). Three-dimensional intact-tissue sequencing of single-cell transcriptional states. Science 361 (6400), eaat5691. 10.1126/science.aat5691 29930089 PMC6339868

[B109] Wood J. I. Wong E. Joghee R. Balbaa A. Vitanova K. S. Stringer K. M. (2022). Plaque contact and unimpaired Trem2 is required for the microglial response to amyloid pathology. Cell Rep. 41 (8), 111686. 10.1016/j.celrep.2022.111686 36417868

[B110] Wu E. Guo X. Teng X. Zhang R. Li F. Cui Y. (2021). Discovery of plasma membrane-associated RNAs through APEX-seq. Cell Biochem. Biophys. 79 (4), 905–917. 10.1007/s12013-021-00991-0 34028638

[B111] Xia C. Fan J. Emanuel G. Hao J. ZHUANG X. (2019). Spatial transcriptome profiling by MERFISH reveals subcellular RNA compartmentalization and cell cycle-dependent gene expression. Proc. Natl. Acad. Sci. U. S. A. 116(39), 19490–19499. 10.1073/pnas.1912459116 31501331 PMC6765259

[B112] Xu L. Lai L. Wen Y. Lin J. Chen B. Zhong Y. (2023). Angiopep-2, an MRI biomarker, dynamically monitors amyloid deposition in early Alzheimer's disease. ACS Chem. Neurosci. 14, 226–234. 10.1021/acschemneuro.2c00513 36599050 PMC9854622

[B113] Xu X. Tao Y. Fu X. Yu T. Li Y. Chen K. (2014). ZIP-Seq: Genome-wide mapping of trinucleotide repeats at single-base resolution. J. Mol. Cell Biol. 6 (1), 93–96. 10.1093/jmcb/mjt048 24334259

[B114] Xue Y. Liu D. Cui G. Ding Y. Ai D. Gao S. (2019). A 3D atlas of hematopoietic stem and progenitor cell expansion by multi-dimensional RNA-seq analysis. Cell Rep. 27 (5), 1567–1578. 10.1016/j.celrep.2019.04.030 31042481

[B115] Yeldell S. B. Yang L. Lee J. Eberwine J. H. DMOCHOWSKI I. J. (2020). Oligonucleotide probe for transcriptome *in vivo* analysis (TIVA) of single neurons with minimal background. ACS Chem. Biol. 15(10), 2714–2721. 10.1021/acschembio.0c00499 32902259 PMC8312762

[B116] Yuan Z. Zhou Q. Cai L. Pan L. Sun W. Qumu S. (2021). SEAM is a spatial single nuclear metabolomics method for dissecting tissue microenvironment. Nat. Methods 18 (10), 1223–1232. 10.1038/s41592-021-01276-3 34608315

[B117] Zhang L. Chen D. Song D. Liu X. Zhang Y. Xu X. (2022a). Clinical and translational values of spatial transcriptomics. Signal Transduct. Target Ther. 7 (1), 111. 10.1038/s41392-022-00960-w 35365599 PMC8972902

[B118] Zhang M. Eichhorn S. W. Zingg B. Yao Z. Cotter K. Zeng H. (2021). Spatially resolved cell atlas of the mouse primary motor cortex by MERFISH. Nature 598 (7879), 137–143. 10.1038/s41586-021-03705-x 34616063 PMC8494645

[B119] Zhang Z. Li Y. Jiang S. Shi F. D. Shi K. Jin W. N. (2022b). Targeting CCL5 signaling attenuates neuroinflammation after seizure. CNS Neurosci. Ther. 29(1), 317–330. 10.1111/cns.14006 36440924 PMC9804050

[B120] Zierhut K. C. Grassmann R. Kaufmann J. Steiner J. Schiltz B. K. (2013). Hippocampal CA1 deformity is related to symptom severity and antipsychotic dosage in schizophrenia. Brain 136 (3), 804–814. 10.1093/brain/aws335 23388407

