# Supplementary material for: Application of spatial transcriptome technologies to neurological diseases
Source: Front Cell Dev Biol. 2023 Mar 3;11:1142923. doi: 10.3389/fcell.2023.1142923 (PMC10020196; doi:10.3389/fcell.2023.1142923)
Supplement: Supplementary file 1 [file Table1.DOCX]

Supplementary Material

Application of spatial transcriptome technologies to neurological diseases

Dongshan Ya ^1,2^, Yingmei Zhang^1,2^, Qi Cui^1,2^, Yanlin Jiang^3^, Jiaxin Yang^1,2^, Ning Tian^1,4^, Wenjing Xiang^5^, Xiaohui Lin^6^, Qinghua Li^1,2,4^, and Rujia Liao^1,2,4*^

*** Correspondence:** Rujia Liao: liaorujia@hotmail.com

# Supplementary Table

**Table 1. A summary of the technical parameters for the mentioned spatial transcriptomic technologies**

| **Method** | **Sample type** | **Approach** | **Spatial resolution** | **Throughput (cells or spots)** | **Gene detection level (depth)** | **Advantages** | **Restrictions** |
| --- | --- | --- | --- | --- | --- | --- | --- |
| LCM-seq | FF or FFPE | Transcriptome-wide | Cellular | 1 | ~10 000 genes | Robust | Low throughput |
| Geo-seq | FF or FFPE | Transcriptome-wide | Cellular | 1 | ~8000 genes | More sensitive than LCM-seq | Low throughput |
| Tomo-seq | FF or FFPE | Transcriptome-wide | Anatomical features | N/A | Whole transcriptome | Construction of 3D profiles | Requires numerous identical biological samples, hence it cannot be applied to human samples |
| TSCS | FF or FFPE | Transcriptome-wide | Cellular | 100 | Whole exome | 10,000 genes can be analyzed | Applied to only 100 cells |
| smFISH | FF or FFPE | Targeted | Subcellular | ~100 | 3 genes | High detection sensitivity | Low throughput |
| seqFISH | FF | Targeted | Subcellular | ~200 | 10~250 genes | Highly multiplex | Need specialized equipment;  Cost increases significantly as the number of targets rises; Limited field of view |
| seqFISH+ | FF | Targeted | Subcellular | 2963 | 10,000  genes | Exceedingly high multiplex | Cost increases significantly as the number of targets rises; Limited field of view |
| MERFISH | FF | Targeted | Subcellular | Tens of  thousands | 100~10,050  genes | Highly multiplex | Need specialized equipment; Cost increases significantly as the number of targets rises |
| split-FISH | FF | Targeted | Subcellular |  | ~300 genes | Reduced false positive rate | High experimental costs |
| FISSEQ | FF or FFPE | Transcriptome-wide | Subcellular | Hundreds | ~8,000 genes | Non-targeted | Low sensitivity  Limited field of view |
| Baristaseq | Cell cultures | Targeted | Subcellular | 206 | ~200 genes | Apply SBS instead of SBL allow read out of up to 15 nt of actual target sequence | A priori chosen targets of limited quantity  Limited field of view |
| STARmap | Fresh or FF | Targeted | Subcellular | ~1000 | ~1 000 genes | Increased sensitivity due to probe-complex, absence of a RT step and tissue transparency | A priori chosen targets of limited quantity;  Limited field of view |
| BOLORAMIS | Cell cultures | Targeted | Subcellular | N/A | 96 genes | Highly multiplex, removes the need for RT, the shortest foot-print needed for detecting transcripts is 25 nt | Spatial crowdedness when targeting many genes, quantify the low level of relative gene expression |
| ST | FF | Transcriptome-wide | 50/100 μm | ~280 | ~5,000 genes | Whole-mRNA analysis | Barcoded regions contain multiple cells; Not single-cell resolution |
| Slide-Seq | FF | Transcriptome-wide | 10 μm | ~70,000 | ~20,000 genes | High resolution | Does not include histology on the same tissue section and has low sensitivity |
| HDST | FF | Transcriptome-wide | 2 μm | ~160 000 | ~17,000 genes | High resolution | Sparse data requires binning, Low sensitivity |
| DBiT-seq | Formaldehyde fixed or frozen | Transcriptome-wide | 10/25/50  μm mosaic grid | N/A | Whole  transcriptome | Flexibility, high spatial resolution co-mapping of proteins | Risk of lateral expansion of barcode |
| Seq-Scope | FF | Transcriptome-wide | 0.5–0.8 μm distant cluster | N/A | Whole  transcriptome | High resolution | Require spatial barcode sequencing |
| Light-Seq | Cell cultures | Transcriptome-wide | <2 µm | 4–1000+ | Whole  transcriptome | Full-transcriptome sequencing based on location, morphology or protein stains, without cellular dissociation; For rare cell populations | the number of addressable regions is currently limited |
| TIVA | Live cells | Transcriptome-wide | Cellular | 1 | ~9000 genes | Can be performed on live cells | Cannot be applied to human tissue |
| Zipseq | Live cells | Transcriptome-wide | Cellular | 0-100 | ~23 000 genes | Can be performed on live cells; Ability to analyze protein; Combinatorial zipcode | Complexity and hybridization efficiency of combinatorial zipcode; Positional details lack |
| APex-seq | Live cells | Transcriptome-wide | Subcellular | N/A | N/A | Can be performed on live cells | Cannot be applied to human tissue |

Fresh frozen samples (FF); Formalin-fixed and paraffin-embedded samples (FFPE), Not available (N/A).

**
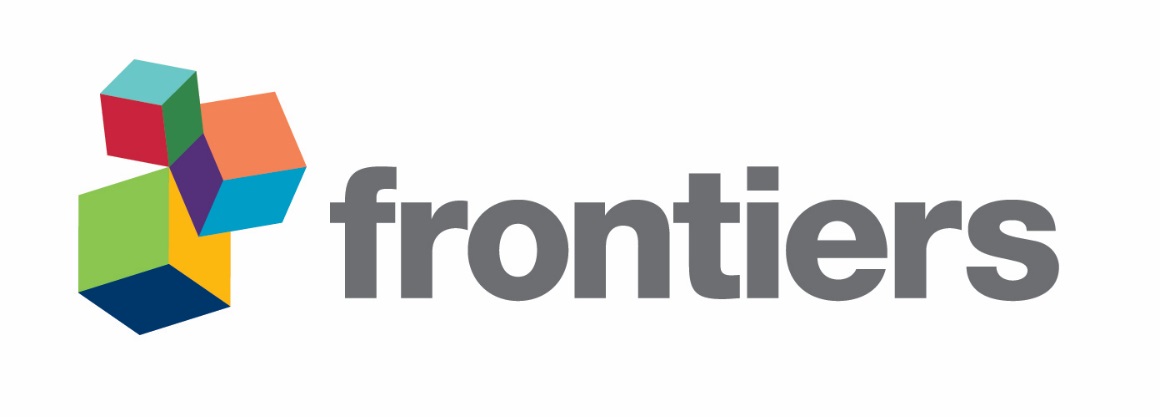
**
